# Role of Vitamins A and D in BCR-ABL *Arf*^*−/−*^ Acute Lymphoblastic Leukemia

**DOI:** 10.1038/s41598-020-59101-4

**Published:** 2020-02-11

**Authors:** Kavya Annu, Cynthia Cline, Kazuto Yasuda, Samit Ganguly, Andrea Pesch, Brittany Cooper, Laura Janke, Monique Payton, Kamalika Mukherjee, Sherri L. Surman, Julia L. Hurwitz, Erin G. Schuetz

**Affiliations:** 1 Department of Pharmaceutical Sciences, St. Jude Children’s Research Hospital, Memphis, TN USA; 2 Department of Pathology, St. Jude Children’s Research Hospital, Memphis, TN USA; 3 Department of Infectious Diseases, St. Jude Children’s Research Hospital, Memphis, TN USA

**Keywords:** Acute lymphocytic leukaemia, Adaptive immunity

## Abstract

The effects of vitamin A and/or vitamin D deficiency were studied in an *Arf*^*−/−*^ BCR-ABL acute lymphoblastic leukemia murine model. Vitamin D sufficient mice died earlier (p = 0.003) compared to vitamin D deficient (VDD) mice. Vitamin A deficient (VAD) mice fared worst with more rapid disease progression and decreased survival. Mice deficient for vitamins A and D (VADD) had disease progression similar to VAD mice. Regulatory T cells, previously shown to associate with poor BCR-ABL leukemia control, were present at higher frequencies among CD4^+^ splenocytes of vitamin A deficient vs. sufficient mice. *In vitro* studies demonstrated 1,25-dihydroxyvitamin D (1,25(OH)_2_VD_3_) increased the number of BCR-ABL ALL cells only when co-cultured with bone marrow stroma. 1,25(OH)_2_VD_3_ induced CXCL12 expression *in vivo* and *in vitro* in stromal cells and CXCL12 increased stromal migration and the number of BCR-ABL blasts. Vitamin D plus leukemia reprogrammed the marrow increasing production of collagens, potentially trapping ALL blasts. Vitamin A (all trans retinoic acid, ATRA) treated leukemic cells had increased apoptosis, decreased cells in S-phase, and increased cells in G_0_/G_1_. ATRA signaled through the retinoid X receptor to decrease BCR-ABL leukemic cell viability. In conclusion, vitamin A and D deficiencies have opposing effects on mouse survival from BCR-ABL ALL.

## Introduction

Vitamin D deficiency (VDD) affects an estimated 1 billion people in the world across all ethnicities and age groups^1–3^. VDD is an independent risk factor for mortality in the general population^4^ and almost 60% of children with malignant diseases have suboptimal vitamin D (VD_3_) levels^5^. Likewise, the world health organization (WHO) estimated 250 million preschool children are vitamin A deficient (VAD), and this increases the risk of disease and death from severe infections. VAD and VDD are not limited to developing countries. Rather, a recent study found that among 45 people tested in Memphis, TN for vitamin A and vitamin D levels, only two individuals had sufficient levels of both vitamins^6^.

Vitamin D is a fat-soluble vitamin that not only regulates calcium absorption and bone metabolism, but can also regulate cell proliferation, differentiation and the immune response. The biologically active form of vitamin D, 1,25(OH)_2_VD_3_, binds to the vitamin D receptor (VDR) that heterodimerizes with the retinoid X receptor (RXR). This complex then binds to VDR-RXR response elements in target genes to regulate transcription. VDR is highly expressed in intestine, kidney and bone, but also in normal and neoplastic hematopoietic cells and mesenchymal stem cells in bone marrow^7^. 1,25(OH)_2_VD_3_ can modify embryonic hematopoietic stem and progenitor cell production^8^. 1,25(OH)_2_VD_3_ inhibits proliferation of mouse and human myeloid leukemia cells^9^ and stimulates myeloid cell differentiation into mature macrophages. Indeed, mice with acute myeloid leukemia (AML) when treated with analogs of 1,25(OH)_2_VD_3_ survived longer than the untreated mice^10^. Moreover, vitamin D insufficiency/deficiency is associated with poor clinical outcome and significantly worse progression free survival of patients from AML^11^, chronic lymphoid leukemia^12^ and non-Hodgkin lymphoma^13^ indicating the clinical significance of vitamin D levels in patients with these types of leukemias and lymphomas.

Acute lymphoblastic leukemia (ALL) is the most common form of childhood cancer accounting for almost 80% of pediatric leukemias^14^, and multiple reports suggest that a majority of leukemia patients are VD_3_ deficient at the time of diagnosis^5,15^. Accordingly, both Dana Farber (ClinicalTrials.gov Identifier number: NCT01574274) and Children’s Hospital of LA (Identifier number: NCT01317940) have ongoing pediatric ALL clinical trials to monitor patients’ VD_3_ levels and to give supplementation to restore sufficiency. However, it is not known how VD_3_ deficiency or supplementation might affect ALL progression or survival. Among the few reports of VD_3_ effects on the B-cell lineage of ALL (B-ALL) (the most common form of pediatric ALL), there have been conflicting results; one study concluded that VD_3_ had no effect on leukemia cell growth in suspension cultures^16^, while another study concluded that VD_3_ inhibited B-cell ALL growth in a clonigenic soft agar assay^17^. Given the controversy in the literature, and the fact that a considerable number of ALL patients are VD_3_ deficient and some of them are receiving VD_3_ supplementation, it is important to test whether VD_3_ levels affect B-ALL cell growth, survival and prognosis.

There are multiple forms of vitamin A, a group of fat-soluble retinoids. The human diet typically includes retinol, retinyl esters, and the provitamin A carotenoids, each of which can be metabolized *in vivo* to its most active metabolite all-trans retinoic acid (ATRA). Retinoids work to regulate cell growth and differentiation and ATRA is now being used to treat some forms of cancers including some leukemias. Retinoids work in part as ligands that activate a number of nuclear receptors (e.g., retinoic acid receptors (RARs), retinoid x receptors (RXRs)) depending on the cell type.

B-ALL is classified into different sub-types based on a number of chromosomal abnormalities, and loss-of function or dominant-negative sequence mutations. Three-to-five percent of pediatric ALL cases and 25% of adult ALL cases, carry the translocation between chromosomes 9 and 22 [t(9;22)] creating the BCR-ABL1 fusion gene (the Philadelphia chromosome (Ph^+^))^18^. At diagnosis around 67% of pediatric Ph^+^ ALL patients also have *Ink4-Arf* deletion (*Arf*^*−/−*^) (hereafter called BCR-ABL ALL or BCR-ABL+ ALL) and approximately 80% have IKAROS (IKZF1) alterations, which are considered hallmarks for high-risk B-ALL^19,20^. Recently it was shown that treatment with retinoids such as ATRA decreased viability of BCR-ABL *Arf*^*−/−*^ and BCR-ABL IKAROS-mutated acute lymphoblastic leukemia *in vitro*^21^, however, it is unknown how vitamin A insufficiency might affect growth of or survival from BCR-ABL leukemia *in vivo*. Therefore, we have tested the effects of vitamin A, D and both A and D sufficiency vs. deficiency on survival in a murine BCR-ABL leukemia model and probed the mechanisms of their differential effects.

## Results

### Vitamin D deficiency improves survival and vitamin A deficiency decreases survival of mice with BCR-ABL^+^ B-ALL

**Figure 1:**
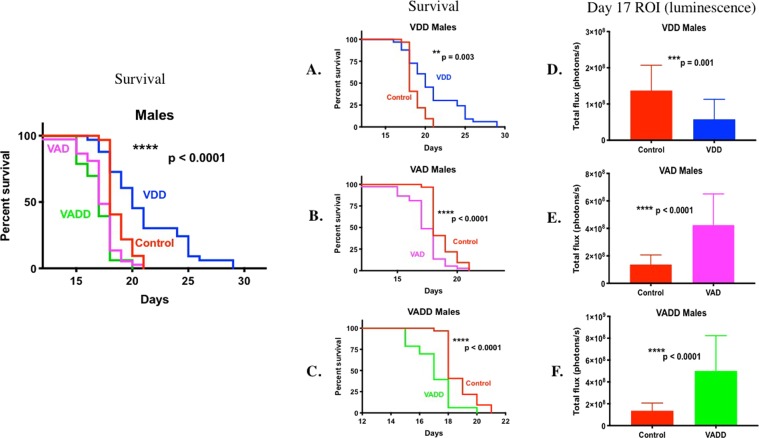
Kaplan Meier survival curves and day 17 leukemia burden of male vitamin sufficient mice (control, n = 34) versus VAD (n = 41), VDD (n = 34), and VADD (n = 35) mice. Kaplan Meier survival curves were plotted for male (**A**) control and VDD mice; (**B**) control and VAD mice; and (**C**) control and VADD mice. (**D–F**) Day 17 BCR-ABL Arf−/− leukemia whole body region of interest (ROI) luminescence in control male mice vs. (**D**) VDD mice; (**E**) VAD mice; and (**F**) VADD mice. The Long-Rank (Mantel-Cox) test was used to find differences between survival curves of control and all vitamin deficient groups (left panel) (****p < 0.0001). The Gehan-Breslow-Wilcoxon test was used to find differences between survival curves of control and each vitamin deficient group (****p < 0.0001, **p < 0.01). The unpaired t-test with Welch’s correction was used to compare leukemia day 17 body burden between each group (****p < 0.0001, ***p = 0.001, **p < 0.01, *p < 0.05).

**Figure 2:**
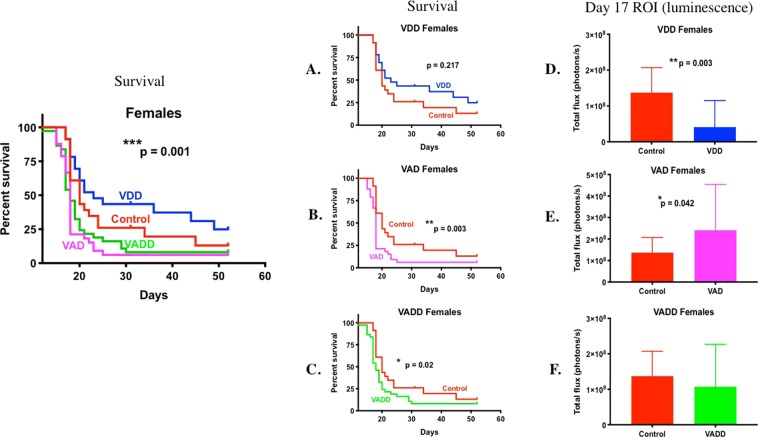
Kaplan Meier survival curves and day 7 leukemia burden of female vitamin sufficient mice (control, n = 27) versus VAD (n = 36), VDD (n = 25), and VADD (n = 38) mice. Kaplan Meier survival curves and day 17 leukemia body burden of female mice with different vitamin levels was analyzed as in Fig legend 1.

Progression of BCR-ABL *Arf*^-/-^ luciferase tagged pre-B leukemia cells and survival from disease were compared between vitamin sufficient control mice and VDD, VAD and VADD mice by *in vivo* bioluminescence imaging of leukemic cells starting on day 8 after injection in male and female mice (Supplementary Figs. S1, S2). Leukemia was detected at day 8 in control, VAD and VADD male mice, but was not detected in the VDD male mice until day 10. By days 14–17 the VAD and VADD mice began succumbing to highly aggressive tumor burden. They had shorter median survival time, consistent with their significantly higher total body disease burden compared to control mice (Fig. 1B,C,E,F, ****p < 0.0001, all studies combined) (Supplementary Fig. S3, individual studies). Surprisingly, compared to VD_3_ sufficient (control) male mice, VDD male mice showed a slower tumor progression over time and had lower average disease burden per mouse (Supplementary Fig. S1, Fig.1D, ***p = 0.001). Consequently, VDD mice survived significantly longer (Fig. 1A, **p = 0.003,) than VD_3_ sufficient control male mice. This can also readily be seen by comparing the proportion of mice surviving on each day after leukemia was administered (Supplementary Table S1). For example, for study 1 on Day 18 only 47% of the control male mice vs. 80.95% of the VDD male mice were surviving; hence the median survival for the control male mice was 18 days versus 21 days for the VDD male mice. Similar effects of vitamin levels on survival from leukemia (shortest to longest: VAD < VADD < Sufficient control < VDD) were observed in female mice (Supplemental Fig. S2 and Fig. 2 all studies combined) (Supplemental Fig. S4, individual studies). Because the growth of disease was slower and median survival times in females were longer than in males, we determined the sex of the murine BCR-ABL leukemia cells using a PCR assay to discriminate X and Y chromosome-specific genes in the leukemia cells. This PCR assay relies on Y chromosome-specific Zfy amplification in males, that is absent in females, and amplification of a 280 bp Y chromosome-specific Sly gene product in males, and a 480/685 bp X chromosome-specific Xlr gene product in females^22^. The BCR-ABL cells were found to be male as indicated by the Zfy and Sly Y chromosomal PCR products (Supplementary Fig. S5).

Vitamins D and A deficiencies were confirmed in serum obtained from terminal blood samples by analysis of 25-OH-VD_3_ in the control, VDD and VADD mice and analysis of RBP levels in the control, VAD and VADD mice (Supplementary Table S2). Complete blood count (CBC) and serum chemistries were compared between control, VAD, VDD and VADD mice with BCR-ABL leukemia and non-leukemic healthy mice (no disease controls (ND)) (Supplementary Tables S3, S4). Compared to leukemic control mice, leukemic male mice deficient for either vitamin A or D, and female mice deficient in vitamin A had decreased red blood cell number, hematocrit and hemoglobin consistent with higher leukemic burden in these mice. Comparing serum chemistries between the groups, the vitamin deficient males had elevated levels of HDL and VAD mice had elevated LDL. No differences between the leukemic control and VDD mice were seen for bone vascularity, trabecular volume, and osteoblast and osteoclast numbers on examination of HE-stained longitudinal sections of tibias. Comparison of mouse body weights (g) on day 0 before leukemia injection showed that only vitamin A deficient body weights differed from controls: male control (21.58 ± 1.95) vs. VDD (21.5 ± 1.99), VAD (20.54 ± 1.86) and VADD (20.35 ± 1.41) mice; and female control (17.83 ± 0.99), VDD (17.09 ± 1.35), VAD (16.55 ± 1.27) and VADD (16.67 + 0.92) mice.

### Bone marrow and spleen BCR-ABL leukemia burden is higher in VAD mice but lower in VDD mice compared to vitamin sufficient mice

To further assess the relative leukemia burden at the primary sites of leukemia replication such as bone marrow and spleen, we quantified the number of luciferase-tagged BCR-ABL cells in the bone marrow (Supplementary Fig. S6A). In addition, a blind scoring of the disease burden in H&E stained histological sections of the hind limb and spleen was performed by the pathologist (Dr. Laura Janke, St. Jude Children’s Research Hospital) (Supplementary Fig. S6B,C). Female mice showed no difference in bone marrow or spleen disease burden between the vitamin sufficient and deficient groups (Supplementary Fig. S6). Consistent with lower total leukemia body burden (Supplementary Fig. S1), VDD male mice had lower levels of bone marrow, hind limb and spleen leukemia (Supplementary Fig. S6A–C). In contrast, despite having higher total leukemia body burden (Supplementary Fig. S1), male mice deficient for vitamin A alone (VAD) and both vitamin A and D (VADD) had significantly lower hind limb leukemia (Supplementary Fig. S6B, ***p = 0.0007, *p = 0.015, *p = 0.045) and lower bone marrow leukemia cells (Supplementary Fig. S6A, *p = 0.016, *p = 0.031), respectively, compared to control mice. This may suggest that in the vitamin A deficient mice, substantial numbers of leukemic cells have moved to the peripheral/systemic blood compartment (not measured).

### Increased frequencies of CD25^+^ FoxP3^+^ CD4^+^ cells in VAD and VADD mice

**Figure 3:**
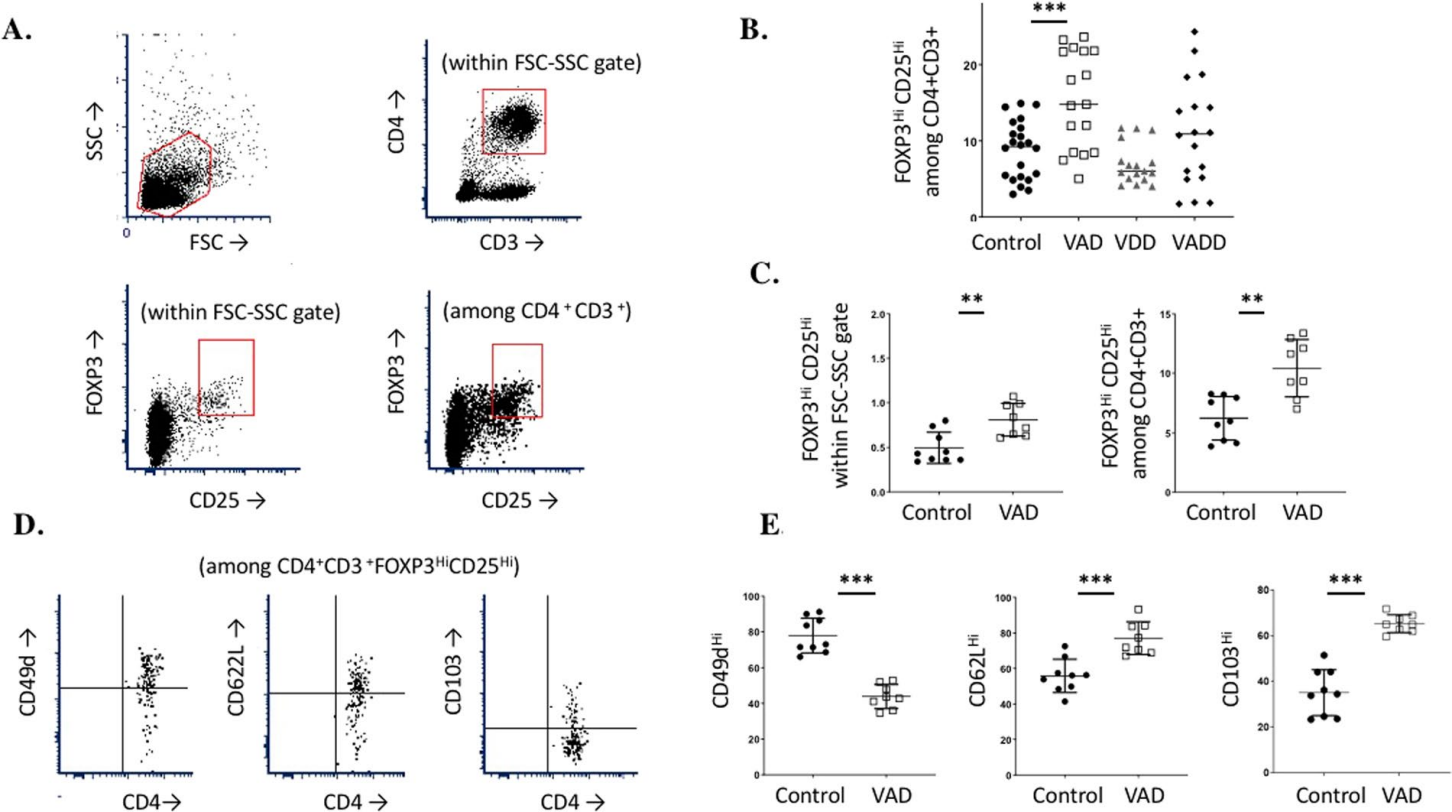
Enhanced Tregs in VAD and VADD mice. (**A**) Flow gating profiles are shown. The lymphocyte gate was selected using FSC-SSC (top left) followed by gating for CD4^+^CD3^+^ cells (top right). FoxP3^Hi^CD25^Hi^ cells were identified within the FSC-SSC gate (bottom left) and within the CD4^+^CD3^+^ gate (bottom right). (**B**) Splenocytes were isolated at the time of sacrifice in tumor studies for each of the four diet groups, and percentages of FoxP3 ^Hi^CD25^Hi^ cells among CD4^+^CD3^+^ cells were determined. (**C**) Control and VAD mice without tumors were tested from two combined experiments for FoxP3^Hi^CD25^Hi^ cell percentages within the FSC-SSC lymphocyte gate (left) and among CD4^+^CD3^+^ cells (right). The total number of FoxP3^Hi^CD25^Hi^ cells per spleen (within the FSC-SSC gate) averaged 3.8 × 10^5^ for control animals and 5.7 × 10^5^ for VAD animals (not shown). Each symbol represents the result from a separate mouse. (**D**) Sample flow diagrams are shown for analyses of CD49^Hi^ cells (left), CD62L^Hi^ cells (center) and CD103^Hi^ cells (right) among the CD4^+^CD3^+^FoxP3 ^Hi^CD25^Hi^ cells in naïve mice. (E) VAD and control mice without tumors were tested from two combined experiments for percentages of CD49^Hi^ cells (left), CD62L^Hi^ cells (center) and CD103^Hi^ cells (right) among the CD4^+^CD3^+^FoxP3 ^Hi^CD25^Hi^ population. Each symbol represents the results from a single mouse. Results were compared using unpaired t-tests (GraphPad Prism, ** indicates p < 0.01, *** indicates p < 0.001).

A previous study^23^ described positive correlations between forkhead box P3 (FOXP3)-positive, CD4^+^ regulatory T cells (Treg) and BCR-ABL *Arf*^*−/−*^ ALL growth. We therefore used flow cytometry to determine how vitamin A and D deficiencies affected these immune cells among splenocytes of test animals at the time of sacrifice. CD25^+^ FoxP3^+^ cell frequencies were significantly higher among CD4^+^ splenocytes in leukemic VAD and VADD mice compared to VDD and control mice (Fig. 3, ***p < 0.001). To determine if the high Treg frequencies were a response to tumor growth, we additionally tested non-leukemic VAD animals. Again, we observed high frequencies of CD25^+^ FoxP3^+^ cells among CD4^+^ splenocytes, and further characterized cells as expressing high CD103, high CD62L, and low CD49d membrane markers.

### Mice that are vitamin D deficient for a shorter time interval still show increased survival from BCR-ABL leukemia compared to vitamin D sufficient mice

All of the mouse survival studies were performed on mice rendered vitamin deficient by placing pregnant females on vitamin A and D deficient diets during pregnancy and parturition and maintaining weaned pups on these deficient diets. Although this diet schedule is necessary to yield vitamin A deficient mice (see methods), because we can generate vitamin D deficiency in a shorter time frame, we compared survival from BCR-ABL ALL between vitamin D sufficient mice and mice rendered VDD by placing them on a vitamin D deficient diet at 4 weeks (weaning) and using them after 10 weeks of age. Despite the shorter duration of vitamin D deficiency, VDD mice still had prolonged survival from BCR-ABL Arf^−/−^ ALL (Supplementary Fig. S7). We are unable to perform similar studies with mice on a VAD diet from weaning since we cannot make the mice vitamin A deficient in this short of a time^24,25^.

### 1,25(OH)_2_VD_3_ increases the number of BCR-ABL ALL cells only when co-cultured with bone marrow stromal cells

**Figure 4:**
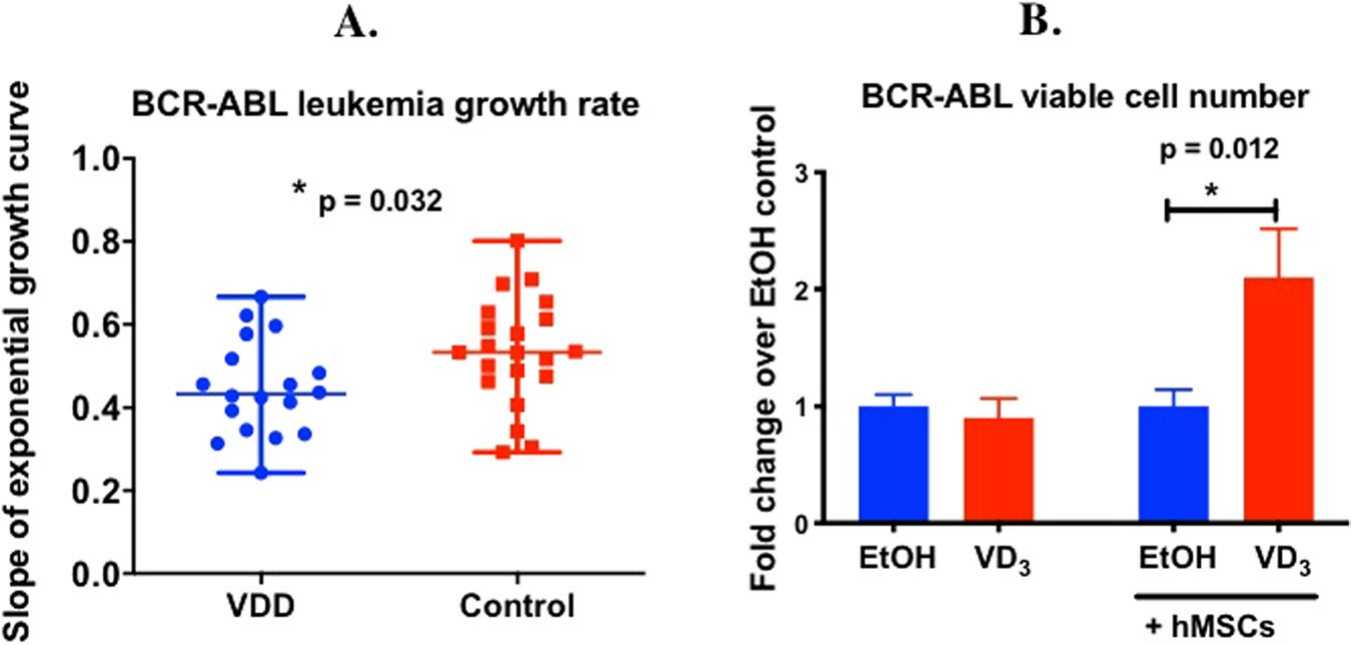
1,25(OH)2VD_3_ increases BCR-ABL ALL cell number *in vivo* and *in vitro*. (**A**) *In vivo* rate of ALL progression was measured by calculating the slope of the exponential growth curve of leukemic cells in VDD (n = 18) vs VD_3_ sufficient (control, n = 22) leukemic mice. (**B**) The effect of 1,25(OH)_2_VD_3_ vs. vehicle treatment on BCR-ABL ALL cell number when cultured in the absence or presence of hMSC was plotted as fold change relative to the control group (n = 3/group). Mann-Whitney nonparametric test on GraphPad was used to determine significance between the groups (*p < 0.05) in 4A, the unpaired t-test was used to compare differences in BCR-ABL cell number in 4B (*p < 0.05).

To begin exploring the mechanism of how BCR-ABL ALL was expanding more rapidly in vitamin D sufficient versus deficient mice *in vivo* (Figs. 1–2, 4A), we examined the effect of 1,25(OH)_2_VD_3_ treatment on BCR-ABL *Arf*^*−/−*^ leukemia cells *in vitro*. Because mouse BCR-ABL^+^ *Arf*^*−/−*^ leukemia cells, like human primary B-ALL cells^17^, lack the vitamin D receptor (VDR) (Supplementary Fig. S8) required for mediating the VD_3_ signal, and because leukemic cells proliferate in the bone marrow, BCR-ABL ALL cells were co-cultured with immortalized human mesenchymal stromal cells (hMSCs) expressing VDR that mimic a bone marrow micro-environment. There was no effect of 1,25(OH)_2_VD_3_ on the number of viable BCR-ABL ALL cells grown without hMSCs (p = 0.5972); however, 1,25(OH)_2_VD_3_ significantly increased the number of BCR-ABL cells (2.102 ± 0.415- fold, p = 0.012) (Fig. 4B) when co-cultured with hMSCs. This result suggests that 1,25(OH)_2_VD_3_ is acting through the stroma to enhance BCR-ABL ALL cell growth. In total, this result showed that 1,25(OH)_2_VD_3_ enhances BCR-ABL ALL cell number *in vitro* and this requires co-culture with bone marrow stroma.

### Increased BCR-ABL ALL migration to 1,25(OH)_2_VD_3_-conditioned hMSCs

**Figure 5:**
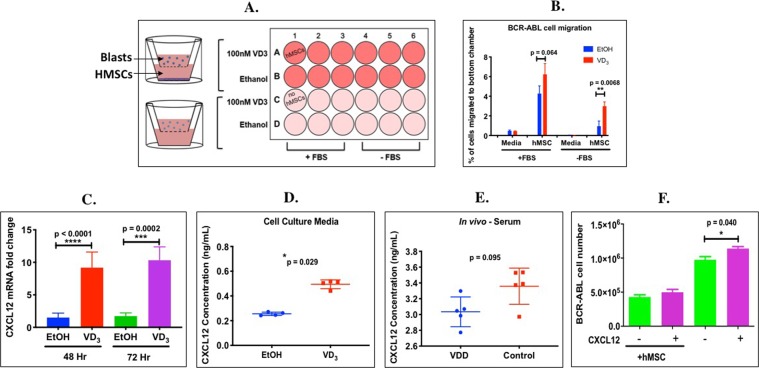
1,25(OH)2VD_3_ increased migration of BCR-ABL leukemia cells to bone marrow stroma and induced *CXCL12* mRNA and protein expression in hMSCs and serum of mice *in vivo* and CXCL12 increased the number of BCR-ABL ALL cells in suspension culture. (**A**) hMSCs were cultured in the receiver compartment of a transwell dish and pretreated 48 hr with either ethanol (vehicle) or 1,25(OH)_2_VD_3,_ n = 3 per group. BCR-ABL ALL LUC^+^ cells were added to the donor compartment and cultured for 24 hr. (**B**) The percentage of the total number of BCR-ABL ALL Luc^+^ cells that migrated to the bottom chamber vs. total number of cells in both compartments was determined. (**C**) Fold change in *CXCL12* (GAPDH normalized) mRNA transcripts levels in hMSC cells treated with 100 nM 1,25(OH)_2_VD_3_ relative to the ethanol (EtOH) control (set to 1), n = 8 or 10 per group. Unpaired t-test was used to determine significance between the groups (**p < 0.01). (**D**) CXCL12 protein expression in the media of hMSC cells treated with ethanol (EtOH) or 100 nM 1,25(OH)_2_VD_3_, n = 4 per group (**E**) CXCL12 protein expression in the serum from VD_3_ sufficient (control) or VDD mice, n = 5 per group. Mann-Whitney nonparametric test on GraphPad was used to determine significance between the groups (****p < 0.0001, ***p < 0.001, *p < 0.05). (**F**) Effect of CXCL12 treatment (48 hr) on the number of viable BCR-ABL LUC cells cultured alone or in co-culture with hMSCs, n = 3 per group. The unpaired t-test was used to compare change in cell number (*p < 0.05).

Since the *in vivo* vitamin D sufficient (control) mice showed increased ALL disease burden in the bone marrow, compared with the VDD mice (Supplementary Fig. S6), we next tested whether this also reflected vitamin D increasing migration of the leukemic blasts to the bone marrow stroma. An *in vitro* cell homing assay was used in which we measured the percentage of BCR-ABL leukemia cells migrating from the donor chamber of a transwell culture through a semi-permeable membrane to the bottom chamber containing either media or hMSC with or without 1,25(OH)_2_VD_3_ conditioning (Fig. 5). There was no effect of 1,25(OH)_2_VD_3_ on migration of ALL cells in the absence of hMSCs. However, a significantly greater percentage of BCR-ABL ALL cells migrated to hMSCs conditioned for 48 hours with 100 nM 1,25(OH)_2_VD_3_ (Fig. 5B, **p = 0.0068). Fetal bovine serum (FBS), which alone can also provide the media with factors required for BCR-ABL ALL migration, further increased the effect of 1,25(OH)_2_VD_3_ (p = 0.064). This result demonstrates that the ability of 1,25(OH)_2_VD_3_ to enhance BCR-ABL leukemic cell migration is not intrinsic to the BCR-ABL blast, but rather, is due to VD_3_-initiated reprogramming of the hMSCs to attract more BCR-ABL ALL cells.

### 1,25(OH)_2_VD_3_ increased CXCL12 mRNA and protein expression in hMSCs *in vitro* and in mouse serum *in vivo*

**Figure 6:**
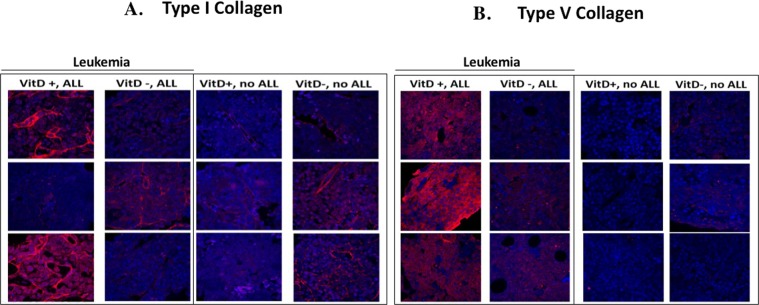
Effect of vitamin D and BCR-ABL leukemia on expression of Type I and Type V collagens in mouse bone marrow. Fluorescent immunohistochemistry was performed on paraffin embedded mouse tibias to examine the expression of collagens I and V *in vivo*. Collagens I and V both show the highest expression in VD_3_ sufficient mice with ALL.

CXCL12 (stromal cell-derived factor-1, Sdf-1) is a chemokine produced by stromal cells, CXCL12 abundant reticular (CAR) cells, endothelial cells and osteoblasts, that plays an important role in normal B-cell lymphopoesis, cell trafficking and homing to the bone marrow^26^, and it has been shown to increase proliferation of primary pre-B-ALL^27^. CXCL12 mRNA (Fig. 5C) expression was higher in hMSCs treated with 1,25(OH)_2_VD_3_ for 48 or 72 hr compared to vehicle-treated cells (9.179 ± 2.415-fold (****p < 0.0001) and 10.320 ± 2.060-fold (***p = 0.0002) respectively). Likewise, cell culture supernatants from hMSCs conditioned for 72 hr with 1,25(OH)_2_VD_3,_ had a 2-fold increase in secreted CXCL12 protein compared with vehicle-treated controls (Fig. 5D, *p = 0.029). There was a trend toward a higher serum CXCL12 protein level in VD_3_ sufficient versus VD_3_ deficient mice (Fig. 6C, p = 0.095). However, we did not find a difference in mRNA expression of the CXCL12 receptor CXCR4 in BCR-ABL ALL cells treated with 1,25(OH)_2_VD_3_ (not shown). Hence, 1,25(OH)_2_VD_3_ reprograms the bone marrow stroma to produce and secrete CXCL12 to attract leukemia cells.

### CXCL12 increases the number of BCR-ABL ALL cells in suspension culture

To determine whether CXCL12 could directly affect BCR-ABL ALL cell number, we treated them in suspension culture with CXCL12. Since VD_3_-stimulated hMSC cells secreted 0.5 ng/ml of CXCL12 into the media (Fig. 5D), BCR-ABL ALL cells in suspension culture, or co-cultured with hMSCs, were treated with 0.5 ng/ml CXCL12 for 48 hr. CXCL12 produced a significant increase (Fig. 5F, *p = 0.04) in BCR-ABL ALL cell number in suspension culture, but no further increase in number when co-cultured with hMSC. This demonstrates that exogenous CXCL12 can directly stimulate growth of BCR-ABL leukemia cells.

### Vitamin D plus ALL reprograms the bone marrow stroma *in vivo*

A growing body of literature demonstrates a role for VD_3_ in remodeling stroma that can then affect cancer growth and chemotherapeutic response^28^. Similarly, the presence of leukemia in the bone marrow has been shown to transform the bone marrow niche into a leukemia-permissive microenvironment^29^. To test whether vitamin D remodels bone marrow extracellular matrix production, immunohistochemistry for type I and V collagen was performed on the bone marrow of healthy vitamin D sufficient and deficient mice, as well as on those with BCR-ABL ALL obtained at the time of sacrifice. Both type I and V collagen were minimally detectable in the bone marrow of healthy mice, regardless of vitamin D levels, but increased in the marrow of VD_3_ sufficient (not VDD) mice with ALL (Fig. 6A,B). Hence, the presence of BCR-ABL ALL together with vitamin D is reprogramming the bone marrow stroma to increase production of type I and V collagens.

### ATRA is signaling through RXR to decrease BCR-ABL ALL viable cell number

**Figure 7:**
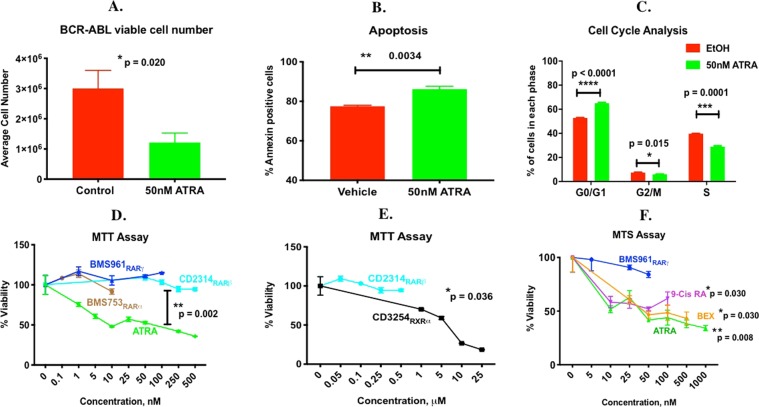
Effect of retinoids and RAR- and RXR-specific agonists on the number of viable BCR-ABL *Arf*^*−/−*^ ALL cells. Effect of 48 hr ATRA or vehicle (control) treatment of BCR-ABL ALL cells in suspension culture on (**A**) BCR-ABL ALL viable cell number, n = 3 per group **(B**) Percentage of apoptotic (Annexin V positive) cells, n = 3 per group; and (**C**) Percentage of cells in each phase of the cell cycle, n = 3 per group. (**D–E**) The number of viable BCR-ABL leukemia cells measured by MTT or MTS assay following 48 hr treatment with various doses of (**A**) ATRA or RARα, β, γ specific agonists, (**E**) RARβ vs. RXRα specific agonist, or (**F**) ATRA, 9-cis-retinoic acid (9-Cis RA), bexarotene (BEX), or RARγ specific agonist, n = 4 per group. The unpaired t-test with Welch’s correction was used to determine significance between the treatments in Fig. 7A,B,D–F as appropriate (**p < 0.01 *p < 0.05).

Since leukemia bearing VAD male mice showed decreased survival (Fig. 1), but a lower bone marrow disease burden (Supplementary Fig. S6), despite a higher level of total body (peripheral) disease (Fig. 1), we first tested whether ATRA treatment differentially affected the number of viable leukemia cells when cultured with or without bone marrow stromal cells. ATRA decreased the number of viable BCR-ABL cells in suspension culture (Fig. 7A) or co-cultured with hMSC (not shown), hence ATRA was directly regulating the BCR-ABL leukemic cells. Subsequent studies were performed on BCR-ABL ALL cells grown alone in suspension culture. BCR-ABL ALL cells treated with ATRA showed increased apoptosis (Fig. 7B), an increased percentage of cells in G_0_/G_1_ but decreased percentage of cells in S-phase of the cell cycle (Fig. 7C). To help identify the nuclear receptor responsible for ATRA’s effect, BCR-ABL ALL cells were treated with pan-agonists for the retinoic acid receptors (RARs) and retinoid X receptors (RXRs), or RARα, RARβ or RARγ or RXRα specific agonists (Table 1) and the number of viable cells determined by MTT and MTS assays. ATRA directly activates RARs and indirectly activates RXR if converted to 9-cis-retinoic acid, an RXR and RAR agonist. BCR-ABL ALL cells express RARs and multiple forms of RXR^21^. In our study, ATRA appears to utilize RXR, and not RAR, to affect the number of viable BCR-ABL ALL cells because RARα, RARβ and RARγ specific agonists all failed to decrease the number of viable leukemic cells, while ATRA, 9-cis-RA, the RXR pan-agonist bexarotene and the RXRα specific agonist potently and efficaciously decreased the number of viable BCR-ABL ALL cells (Fig. 7D–F). Hence, dietary vitamin A is important in directly modulating the number of viable BCR-ABL ALL cells *in vivo* utilizing the RXR.

**Table 1:** Retinoic Acid Receptor and Retinoid X Receptor ligands tested.

Agonist & Receptor selectivity	Concentrations used	Receptor Kd or EC50
BMS753; RARα	0–10 nM	2 nM
CD2314; RARβ	50–500 nM	145 nM
BMS961; RARγ	1–100 nM	30 nM
CD3254; RXRα	1–25 µM	1.5–2 µM
ATRA; RARs	1–1000 nM	
9-cis-retinoic acid; RARs/RXRs	0–10 nM	8–15 nM (RXRs); 92–148 nM (RARs)
Bexarotene; RXRα, β, and γ	0–500 nM	28, 25, and 20 nM

## Discussion

Despite the high frequency of vitamin A and/or D deficiencies in humans worldwide, this is the first study to test and demonstrate that host deficiencies in vitamins A and D can affect growth of BCR-ABL ALL *Arf*^*−/−*^ leukemia and survival of mice from this disease. Our study found mice deficient in vitamin A had markedly accelerated disease progression and decreased survival from BCR-ABL ALL compared to mice that were vitamin A sufficient. Surprisingly, vitamin D deficiency had the opposite effect - slowing growth and enhancing survival time from the leukemia.

Although recent work demonstrated that therapeutic ATRA treatment of BCR-ABL ALL cultured cells significantly decreased their viability if the leukemic blasts also had *IKZF1*-mutated or *Arf* nullizygous alleles^21^, it was not clear whether a deficiency of host vitamin A would have such a detrimental effect on the host survival from BCR-ABL *Arf*^*−/−*^ leukemia. The effect of ATRA on BCR-ABL ALL viability did not require co-culture with bone marrow stromal cells but appeared to be intrinsic to the leukemic cell and vitamin A appeared to signal via RXR. Nevertheless, it was notable that the level of whole-body leukemia, but not bone marrow or spleen leukemia, was significantly greater in the vitamin A deficient vs. sufficient mice (Supplementary Fig. S1 vs. Fig. S6). This may be due to the known changes in bone marrow of VAD mice, including alterations in the bone marrow microenvironment^30^ and cell and metabolite profiles^31,32^.

The difficulty in rendering mice vitamin A deficient required that we place pregnant females on VAD diet during gestation, after parturition, and continue the pups on the same VAD diet after weaning to successfully produce VAD mice at six weeks of age (see Methods). To maintain uniformity between the vitamin deficient groups, the same method was used to generate mice deficient for both vitamins. Since vitamins do influence many organ systems of the mouse it is possible that deficiencies of either vitamins on those systems during pregnancy could have affected the ALL outcomes in this study. When vitamins A and D are lacking in the diets of mothers and progeny, protective responses may be influenced both in terms of structural barriers and adaptive immunity. Vitamin deficiencies can alter hematopoiesis as well as mature lymphocyte activation potentials^33,34^. Moreover multiple studies report that vitamin A deficiency may result in anemia and altered serum iron levels (in rats)^35^. Although serum iron levels (µg/dl) in representative non-diseased vitamin sufficient mice (161.3 ± 18.27) were not different from representative non-diseased VDD (185.3 ± 35.81), VAD (187.5 ± 62.47) and VADD (132.80 + 64.31) mice, we cannot rule out an influence of vitamin deficiencies during gestation on hematopoiesis. However, a similar effect of vitamin D deficiency on survival from BCR-ABL ALL was seen regardless of whether mice were exposed to a VDD diet during gestation (Fig. 1), or started the diet at weaning (Supplementary Fig. S7), demonstrating the effect vitamin D levels on BCR-ABL leukemogenesis in this mouse model does not require vitamin D deficiency during gestation.

Deficiency of vitamin A appears to also have negatively affected the host adaptive immune response to eradicate BCR-ABL ALL. Vitamin A deficient mice, both with and without tumors, exhibited higher Treg FoxP3^+^CD25^+^ cells among CD4^+^ splenocytes compared to controls. These results were consistent with studies of Manlove *et al*.^23^ showing that one mechanism by which BCR-ABL leukemia cells escaped an antileukemia T cell response was by supporting conversion of cells to a Treg phenotype. Given that Treg frequencies were already higher in VAD mice compared to controls, it follows that VAD mice were particularly vulnerable to tumor escape. We further identified cells as displaying low CD49d, high CD103, and high CD62L compared to controls. Tregs with low CD49d have been associated with the highest suppressive activity^36,37^. CD103 and CD62L are each homing markers, instrumental in cell trafficking and tissue residence^38–40^. We propose that the abnormal levels of homing markers on Tregs in VAD mice helped sustain high Treg numbers and inhibitory functions in the spleen and peripheral blood, further supporting tumor growth. Our data fine-tune the common assumption that vitamin A drives FoxP3^+^ Treg development while down-regulating Th17 cells and immune responses. In contrast to dogma, Xiao *et al*. found that vitamin A did not upregulate Treg when mice suffered from inflammation in a model of experimental autoimmune encephalomyelitis^41^, and our lab has demonstrated that VAD mice are characterized by frequent FoxP3^+^ CD25^+^ T cells and a reduced adaptive immune response^38,42–44^. Although mice have been described as being resistant to weight loss due to vitamin A deficiency^25^, we observed that VAD and VADD mice had lower body weight on day 1 of our study compared to vitamin sufficient mice. This emphasizes that VAD and VADD mice suffer from several influences of vitamin deficiency including those that affect immune system function and repair^45^. The animals’ reduced adaptive immune responses may lead to early death, as we observed in our leukemia study. Possibly, the high Treg frequencies among CD4^+^CD3^+^ lymphocytes contributed to the poor outcome of VAD and VADD mice.

Surprisingly, host VD_3_ sufficiency vs. deficiency was detrimental to host survival from BCR-ABL *Arf*^*−/−*^ ALL. Importantly, 1,25(OH)_2_VD_3_ had no effect on growth of the leukemia cells in suspension culture but required co-culture with the bone marrow stroma. This result is consistent with the apparent absence of the VDR in mouse BCR-ABL^+^ ALL *Arf*^*−/−*^ leukemia cells, and similarly its absence in human primary B-ALL cells^17^. Our results also offer a cautionary reminder for the many studies that test the effect of chemicals on leukemia cells grown in suspension culture, without co-culture of bone marrow stromal cells, as these studies may miss important leukemia cell responses that are only seen in a co-culture setting. Bone marrow stroma cells are known to form a microenvironment not only conducive for leukemic cell growth, but that can also regulate responsiveness to anti-leukemic therapies such as asparaginase and tyrosine kinase inhibitors^46,47^. Our results extend this paradigm to the effect of 1,25(OH)_2_VD_3_ on growth and migration of BCR-ABL^+^ ALL *Arf*^*−/−*^ leukemia cells.

1,25(OH)_2_VD_3_ actively induced bone marrow stroma to release CXCL12 *in vitro*, and increased CXCL12 serum levels *in vivo*, resulting in an increased ability to attract the BCR-ABL^+^ *Arf*^*−/−*^ ALL to the marrow and support its growth. Other cells in the bone marrow, but absent from the hMSC, that produce CXCL12 may also be responsive to 1,25(OH)_2_VD_3_ and increase production of the chemokine CXCL12. For example, perivascular CAR cells (CXCL12 abundant) are known to increase homing of hematopoietic stem cells to the perivascular niche where they actively proliferate and we have preliminary data showing that bone and bone marrow CAR cells are significantly greater in number in vitamin D sufficient vs. deficient mice (K. Annu, unpublished data).

*In vivo*, vitamin D, in the presence of BCR-ABL^+^ *Arf*^*−/−*^ leukemia, also remodeled the bone marrow stroma, inducing expression of Type 1 and V collagens. Others have reported that increased bone marrow reticulin and collagen fibers are associated with malignant diseases and may anchor and trap the leukemic cells in the marrow providing an environment where they become more drug resistant^48^. While this study probed the interaction of 1,25(OH)_2_VD_3_ signaling through the co-cultured bone marrow stromal cells, *in vivo* multiple other cell types in the bone marrow express VDR, including osteoblasts. Hence*, in vivo*, vitamin D may signal through VDR in additional cells in the bone and bone marrow influencing the microenvironment and differentially altering the growth and viability of the leukemia cells.

It will be important to determine whether the finding that vitamin A and D levels significantly affect the growth of BCR-ABL^+^ *Arf*^*−/−*^ ALL in mice *in vivo* can be extended to other subtypes of pediatric leukemia. Moreover, do these results in mice translate to humans? Interestingly, a study in children that measured dietary vitamin D intake in newly diagnosed children with ALL found that vitamin D intake was significantly different between patients who presented with high-risk ALL vs. standard-risk ALL. Failure to meet daily dietary amounts of vitamin D was higher in the standard risk vs. high-risk patients, while the proportion of patients who met the vitamin D dietary requirements was higher in the high risk vs. standard risk patients^49^. Clearly our findings suggest that the clinical levels of both vitamins A and D should be measured in leukemic patients over the course of therapy to determine if there is an association in humans between vitamin sufficiency and survival from different subtypes of leukemia, and if vitamin supplementation to vitamin deficient patients affects survival from acute lymphoblastic leukemias.

## Materials and Methods

### Animals

C57BL/6J, day 4–5 estrus pregnant (E4–5) female mice were obtained from Jackson Laboratories (Bar Harbor, ME, USA). Animals arriving at the St. Jude Children’s Research Hospital (SJCRH) animal facility were placed on Purina Mills (Purina Mills, St. Louis, MO, USA) diets – either control (cat. no. 5W9M contained 1.5 IU of vitamin D and 15 IU/g of vitamin A palmitate), VDD (cat. no. 5W9X, contained 0 IU of vitamin D and 15 IU/g of vitamin A palmitate), VAD (cat. no. 5WA2 contained 1.5 IU of vitamin D and 0 IU/g of vitamin A palmitate), and VADD (cat. no.5WA5, contained 0 IU of vitamin D and 0 IU/g of vitamin A palmitate) were used for the survival and other *in vivo* studies. Pregnant dams were maintained on these diets from the time of arrival, throughout the pregnancy, and after parturition. At weaning mouse pups were maintained on their respective diets. The rationale for this schedule to obtain vitamin A deficient mice is because multiple studies showed the difficulty of rendering vitamin A deficiency in mice. Depriving weanling mice of vitamin A does not yield, or at least takes a year to yield, vitamin A deficiency in mice^24^. This ability of mice to withstand long periods of vitamin A deprivation has been attributed to initial large stores of vitamin A (from parturition) at weaning^25^. Placing pregnant females on a vitamin A deficient diet during gestation beginning at day E5, after parturition, and continuing the same diets after weaning produced successful vitamin A deficiency at 6 weeks of age^50^. Hence, we used this strategy since it ensured that test animals would be vitamin A deficient prior to reaching an advanced age. Furthermore, to maintain uniformity between all vitamin deficient and sufficient groups we used the same strategy to generate the vitamin A and D deficient mice.

However, it is possible to generate vitamin D deficiency in mice by transitioning them to a vitamin D deficient diet at weaning. This would eliminate any potential effect of a vitamin D deficient diet on the status of any other systems in pups derived from pregnant mothers put on VDD diets at pregnancy day 5. Hence, mice in a separate set of survival studies, were placed at weaning on a VDD diet (Harlan catalog no. 5A69, 0 IU of vitamin D) and vitamin D sufficient control diet (catalog. no. 5BV8, 3.3 IU of vitamin D).

Serum 25-OH VD_3_ levels were routinely measured from representative mice in the VDD group (starting at 4 weeks of age) to determine vitamin D status. A level of 20–50 ng/mL is considered vitamin D sufficient; 12–20 ng/mL is vitamin D insufficient, and less than 12 ng/mL is vitamin D deficient. Sera were sent to Michigan State University (Lansing, MI, USA) for 25-OH-VD_3_ testing. Serum from representative mice in the VAD and VADD groups were tested for retinol binding protein (RBP) (R&D Systems, Mouse RBP4 Quantikine ELISA Kit, Cat # MRBP40) levels (starting at 6 weeks age) as an indicator of vitamin A status. RBP values were less than 5,000 ng/mL in all tested VAD and VADD mice. Serum 25-OH VD_3_ and RBP levels were also measured in mice at the terminal time point in the leukemia survival studies.

### Ethical approval statement

Association for Assessment and Accreditation for Laboratory Animal Care (AALAC) guidelines and Institutional Animal Care and Use Committee (IACUC) approved protocols were followed for all the experimental procedures and housing of the mice. All methods were carried out in accordance with the relevant guidelines and regulations.

### Leukemia and bone marrow stroma cells

Murine BCR-ABL^+^ *Arf*^*−/−*^ cells tagged with luciferase (Luc^+^) or green fluorescent protein (GFP^+^)^51^, created by Dr. Charles Sherr (St Jude Children’s Research Hospital) and provided by Dr. Mary Relling (St. Jude Children’s Research Hospital), were cultured as previously described^52^. Human mesenchymal stromal cells (hMSCs, bone marrow stroma) immortalized with telomerase^53^ were created by Dr. Dario Campana and provided by Dr. Jun Yang (St. Jude Children’s Research Hospital), and cultured as described^53^.

### BCR-ABL ALL disease generation and monitoring

On day zero, 2,000 BCR-ABL *Arf*^*−/−*^ Luc^+^ cells were injected retro-orbitally into syngeneic, unconditioned 6–10-week-old mice, alternating injection order between cages and groups. The first experiment was run with control male (M) (n = 17), VDD M (n = 23), VAD M (n = 31), VADD M (n = 27) and control female (F) (n = 14), VDD F (n = 14), VAD F (n = 28), VADD F (n = 31) mice. Mice were anesthetized with isoflurane and images using Xenogen IVIS-200 (Caliper Life Sciences, Hopkinton, MA) were acquired post-intraperitoneal injection of 200 µL of 100 mg/kg D-Luciferin (Caliper Life Sciences, Hopkinton, MA). The bioluminescent signal (photons/s) was acquired from a fixed region of interest (ROI) starting on day 8 after leukemic cell injection and repeating every 3–4 days to monitor disease progression. Acquired images were analyzed with Living Image 3.1 software (Caliper Life Sciences). BCR-ABL-ALL Luc^+^ disease burden was quantified from whole animal total luciferase flux measurements (photons/s) from the images. Moribund state of mice was determined based on hind limb paralysis, a scruffy coat, lethargy, or an inability to obtain food or water. At this time before euthanasia, blood was collected for the analysis of cholesterol, triglycerides, high-density lipoprotein (HDL) and low-density lipoprotein (LDL), white blood cell number, and serum for 25-(OH)VD_3_ and RBP levels. Spleen was harvested. Bone marrow cells were flushed from the femur using a needle with either cell culture media or RNAlater (Invitrogen by ThermoFischer Scientific, Vilnius, LT). The cells were transferred to a 1.5 mL Eppendorf tube and pelleted, the cell pellets were resuspended in 100 µL PBS, and transferred to white, clear-bottomed 96-well plate (Costar, Corning Inc., Kennebunk, ME, USA) to which 100 µL of Bright-Glo™ (Promega, Madison, WI, USA) was added. A standard curve with a range of 3,000 to 2 million BCR-ABL cells was used in order to convert the luminescence value into the number of BCR-ABL cells per well. Luminescence was measured using the BioTek Synergy™ 4 Hybrid Microplate Reader. A portion of spleen and hind limb were individually fixed in 10% formalin; 24 hr later the tissues were transferred to Cal-Rite (Thermo Scientific, Pittsburgh, PA) for a minimum of 48 hr. Tissues were embedded in paraffin, and 4 µm sections were cut and stained with hematoxylin and eosin. Slides were evaluated by a pathologist to determine the extent of leukemia burden, scoring the tissues on a 6-point scale from 0–5. The entire experiment was repeated, and both studies were combined for the data analysis with a total of control M (n = 34), VDD M (n = 34), VAD M (n = 41), VADD M (n = 35) and control F (n = 27), VDD F (n = 25), VAD F (n = 36), VADD F (n = 38) mice. To measure bone marrow vascularity and trabecular volume, the slides were scanned using an Aperio ScanScope (Aperio Technologies, Vista, CA) with a 20X objective. Measurements were performed using ImageScope software. The metaphyseal region defined as beginning at the physis and ending 1 mm distal to it; this marrow area was outlined to obtain total metapyseal area. For measurement of bone marrow vascularity all vascular lumens (sinusoids) were outlined within that area. Two types of data were calculated from these measurements, % vascular area (um^2^ vessel lumen/um^2^ marrow), and length (um vessel/um^2^ marrow). For trabecular volume, all trabeculae were outlined within this area and the output was calculated as % trabecular area (um^2^ trabecular bone/um^2^ marrow). Osteoblast and osteoclast numbers, as well as leukemia burden, were evaluated semi-quantitatively by a veterinary pathologist (L.J.J.) using a 5-point scale.

### PCR to determine gender of BCR-ABL ALL cells

The gender of the murine BCR-ABL^+^ *Arf*^*−/−*^ cells was determined by PCR of genomic DNA for the presence of X and Y-chromosome specific markers^22^. DNA was amplified with the following primer pairs: Sly/Xlr_F, 5′-GATGATTTGAGTGGAAATGTGAGGTA-3′; Sly/Xlr-R, 5′-CTTATGTTTATAGGCATGCACCATGTA-3′ and Zfy_F, 5′-GAC T A GACATGTCTTAACATCTGTCC-3′ and Zfy_R, 5′-CC T A TTGC ATGGACTGCAGCTTATG-3′ and amplification conditions were as described^22^.

### Analysis of *in vivo* growth of leukemia

Change in leukemia burden with time was analyzed from the change in whole body luminescence data. Luminescence data for every mouse was normalized with its baseline luminescence value from day 8. Fold change in luminescence over baseline was plotted against days, which was assumed to follow the exponential growth equation, $Fold\,change\,in\,luminescence=A\,{e}^{B\ast t}$, where, A is the intercept and B is the slope of the exponential growth curve. Parameters A and B were estimated by fitting the fold change in luminescence vs. days for each mouse using Microsoft Excel-based trendline analysis. Correlations were considered acceptable only if r^2^ > 0.7. All the survivors were removed from the analysis as there was not measurable leukemia in these mice. Other filtration criteria were: (1) fold change in luminescence has to be > 1, i.e., increase in luminescence over the baseline, (2) positive correlation of fold-change in luminescence with days, (3) there have to be at least three data points for the assessment of correlation, and (4) since the same number of leukemic cells were injected into the mouse, we expected to have no significant difference in intercept A and thus outlier intercepts were removed from the analysis. After filtration of the data, individual slopes (B) were computed for each mouse and compared between the groups, data represented as median slope ± range and Mann-Whitney test at p < 0.05 was used to analyze the significance.

### BCR-ABL ALL viable cell number assay

Luciferase-tagged BCR-ABL cells were plated at a density of 500 cells per well on a 24 well plate and treated with either 1,25(OH)_2_VD_3_ (50 or 100 nM) or ethanol (vehicle) and the plates incubated at 37 °C 8% CO_2_. We tested the effect of a range of 1,25(OH)_2_VD_3_ concentrations on the BCR-ABL ALL viable cell number and found no effect with 1–10 nM, but equal effectiveness of 50–100 nM. 100 nM 1,25(OH)_2_VD_3_ was chosen for experiments for the following reasons: First, since the duration of our 1,25(OH)_2_VD_3_ studies was routinely 48 hr (but as long as 96 hr), and the vitamin is not replenished after dosing at zero hr, and 1,25(OH)_2_VD_3_ has a half-life of 15 hr, the effective concentration remaining at the end of a 48 hr experiment following a 100 nM treatment is estimated to be 25 nM. In humans the concentration of 1,25(OH)_2_VD_3_ in human plasma is 30 pg/ml (0.072 nM), but the *bone marrow concentration* is 500-fold higher (36 nM)^35^. Alternatively, the cells were plated at 125,000 cells/mL and treated for 48 hrs with ATRA (50 nM). To measure cell number, the entire contents of each well were transferred to a 1.5 mL Eppendorf tube, the cells were pelleted, resuspended in 100 µL PBS, and transferred to white clear-bottomed 96-well plates (Costar Corning Inc., Kennebunk, ME, USA) and 100 µL of Bright-Glo™ (Promega, Madison, WI, USA) was added. Luminescence was read using the BioTek Synergy™ 4 Hybrid Microplate Reader. Standard curves were prepared in triplicate using known numbers of Luc^+^ BCR-ABL cells, then the number of BCR-ABL cells in experimental samples was determined by interpolation, and results plotted of average cell number/treatment group or fold differences between vitamin treated and vehicle-treated wells.

### Cell cycle analysis

BCR-ABL-ALL-gfp cells in suspension culture were treated with DMSO and ATRA for 48 hr, harvested, and stained with annexin/DAPI (4′,6-diamidino-2-phenylindole). The cells were pre-treated with RNAse (DNAse free) and suspended in a hypotonic propidium iodide (PI) fluorescent dye solution to quantitatively stain the DNA in cell nuclei of CD45^+^ cells. The stained cells were filtered through 40 μm-diameter mesh to remove clumped nuclei and sorted by fluorescence-activated cell sorting (FACS). The percentage of cells in each cell cycle phase i.e., G_0_/G_1_, S, G_2_/M was analyzed using BD Biosciences FACSDIVA^TM^ software.

### Immune cell phenotyping by cytometry

Flow cytometry was performed at the time of mouse sacrifice in tumor experiments (mice were 6–10 weeks old when tumor cells were first administered), and additionally on naïve, non-tumor bearing mice (>6 months of age). Spleen cells were suspended using a 70 μM cell strainer (Corning Falcon^TM^, Durham, NC, USA). Cells were pelleted and red blood cells were lysed using red cell lysis buffer (Stem Cell 07850 Canada). Spleen cells were counted using a Biorad TC20 automated cell counter. Approximately 1 million cells were added per well in a 96 well round bottomed plate and pelleted. Pellets were flicked and vortexed to suspend cells. Antibody cocktails were added with 40 μl/well for a 15 min incubation on ice in the dark. Each antibody was used at a 1:100 dilution for the staining of membrane markers. Plates were washed with staining wash buffer (SWB, phosphate buffered saline [PBS] with 1% fetal calf serum [FCS]). For subsequent intracellular FOXP3 staining, cells were fixed and permeabilized with the FOXP3 Staining Buffer Set (Cat# 00-5523-00, eBioscience). Anti-FOXP3 antibody (diluted 1:100, eBioscience) was added in Perm Buffer from the Buffer Set for 15 min. Cells were washed and suspended in 100 μl SWB for analyses. Stains from Biolegend or eBioscience in one combination included anti-CD3 (145-2C11, BV711, Cat #100349), anti-CD4 (GK1.5, BV421, Cat# 100443), anti-CD8 (53.67, AF700, Cat# 100730), anti-CD25 (PC61, APC, Cat# 102012), and anti-FOXP3 (PCH101, PE, Cat# 12-4776-41). An additional cocktail used to study splenocytes in naïve mice included (but was not limited to) anti-CD4 (DAPI), anti-CD8 (APC Alexa 700), anti-CD3 (BV711), anti-CD25 (PE-Cy7), anti-FOXP3 (PE), anti-CD62L (BV605), anti-CD49d (PE-Texas red), and anti-CD103 (FITC). Cells were analyzed using an LSRFortessa X-20 (BD Biosciences) and FCS Express Software.

### *In vitro* homing assay

hMSC (100,000/well) were plated onto the bottom of 24-well plates and treated with ethanol vehicle or 100 nM 1,25(OH)_2_VD_3_ for 48 hours with half of the wells/group treated with media lacking fetal bovine serum (FBS). Identical 24-well plates without hMSC were prepared. Then 6.5 mm Transwell Permeable Supports with a 3 µm polycarbonate membrane (Costar Corning Inc., Kennebunk, ME, USA) were added to each well and 1 million BCR-ABL luciferase(luc)^+^ cells in 100 µL of media was added to top donor compartment and incubated at 8% CO_2_ for 24 hr (Fig. 3A). The bottom of each transwell membrane was then washed with media to collect any attached cells. A cell scraper was used to remove all cells from the wells, which were pelleted and resuspended in 100 µL PBS and 100 µL of Bright-Glo™ (Promega, Madison, WI). The BioTek Synergy™ 4 Hybrid Microplate Reader was used to read the luminescence on a white, clear-bottom 96 well plate (Costar, Corning Incorporated, Kennebunk, ME, USA). Luminescence from 3,000-2 million BCR-ABL ALL luc^+^ cells was used to generate a standard curve and the relative BCR-ABL ALL cell number in the receiver compartment was interpolated from the standard curve which was used to determine the percentage of total cells that had migrated from the donor to the receiver compartment.

### mRNA quantitation by real-time PCR

A confluent layer of human bone marrow mesenchymal stem cells (hMSCs) immortalized with telomerase^53^ were treated with vehicle or 100 nM 1,25(OH)_2_VD_3_ for 48 or 72 hours. RNA was extracted using Trizol (Ambion Life technologies, CA, USA or Thermo Fisher Scientific, CA, USA) and the Qiagen RNEasy clean-up kit (Hilden, Germany). 500 ng of RNA was used to create cDNA using the ThermoScript™ RT-PCR System (Invitrogen ThermoFischer, CA, USA) that was then diluted to a total volume of 60 µL. Primers for amplification of human CXCL12/SDF-1αwere (F) 5′-AGAACTGTTGGCAAGGTGACA-3′ and (R) 5′-CTGACATTCATATGGCTCTCATTC-3′; and for mouse CXCL12/SDF-1αwere (F) 5′–CAGTGACGGTAAACCAGTCAGC- 3′ and (R) 5′–TGGCGATGTGGCTCTCG- 3′. Real-time PCR samples were run at 95°C for 15 minutes and then 40 cycles of 92°C for 30 sec, 60°C for 30 sec, and 68°C for 1 min followed by a dissociation step. CXCL12 mRNA expression levels were determined from the averaged Ct values by the comparative Ct method. CXCL12 mRNA values were normalized to GAPDH mRNA in the same sample to control for RNA quality.

### PCR to detect VDR mRNA

The expression of mouse VDR (mVDR) mRNA in BCR-ABL ALL cells and mouse duodenum and kidney, and of human VDR (hVDR) mRNA in human LS180 intestinal cells and hMSCs was determined by PCR. VDR and control GAPDH mRNAs were amplified from cDNA using the following primer pairs:

mVDR-F, CTCCTCGATGCCCACCACAAGACCTACG;

mVDR-R, GTGGGGCAGCATGGAGAGCGGAGACAG;

hVDR-F, CGGCCGGACCAGAAGCCTTT;

hVDR-R, CGGGGCACGTTCCGGTCAAA; GAPDH-F, ACCACAGTCCATGCCATCAC; GAPDH-R, TCCACCACCCTGTTGCTGTA and Promega PCR master mix (catalog number M7505). Following 95°C for 2 min, mVDR and hVDR were amplified by cycles of denaturation at 95°C for 30 sec, annealing at 60°C for 30 sec, extension at 72°C for 30 sec; and extension at 72°C for 5 min; 4°C hold. The product was mixed with 6x DNA loading dye (Promega, WI, USA) and run on a 2% agarose gel (used SYBR Safe DNA gel stain) at 100 V for 90 minutes. Images were taken using Image Lab Software^TM^.

### CXCL12 ELISA assay

The Quantikine ELISA Kit (R&D Systems, Minneapolis, MN, USA) for human CXCL12/SDF-1α was used to assay both cell culture media and mouse serum according to the manufacturer’s protocol. A standard curve was generated using the included controls and used for comparison to a positive control as well as the unknown samples. Cell culture media samples (n = 3) from hMSCs in a 6-well dish were assayed undiluted after 48-hour treatment with either ethanol or 100 nm 1,25(OH)_2_VD_3_. Undiluted serums from mice on either a VD_3_ sufficient (control) or VD_3_ deficient diet (VDD group; n = 5 per group) were also assayed using this approach. The BioTek Synergy™ 4 Hybrid Microplate Reader was used to measure fluorescence at 450 nm and 540 nm. Final values used for comparison were determined by subtracting the reading at 540 nm from the reading at 450 nm; this step was designed to correct for optical imperfections in the plate.

### Immunohistochemistry

VDD and control mice were perfused with 4% paraformaldehyde and the hind limbs were fixed in 10% neutral buffered formalin, decalcified, embedded in paraffin, sectioned (4 *μ*m) and placed onto glass slides for staining. Antibodies for IHC were obtained from Abcam and diluted in Phosphate Buffer Saline with 0.3% Triton X-100 (PBST). Slides containing either mouse tibia or mouse hind limb were deparaffinized and antigens were retrieved using Target Retrieval solution pH 6.0 (Dako, Carpinteria CA, USA) in a pressure cooker for 15 min. After retrieval, slides were rinsed with PBST, treated with 3% hydrogen peroxide, and blocked with Background Sniper (Biocare Medical, Pacheco, CA, USA). Slides were incubated overnight at 4 °C with primary specific antibodies (rabbit anti-collagen I, ab34710 (1:500) and rabbit anti-collagen V (ab7046) (1:50), or the appropriate isotype control antibody, rinsed twice with PBST, and incubated with the secondary antibody (Donkey anti-rabbit 568, ab10042) for 2 hr. Slides were rinsed again in PBST and mounted using ProLong® Gold Antifade Mountant with DAPI (Molecular Probes by Life Technologues^TM^, Eugene, OR, USA) and allowed to dry overnight before viewing. Images were obtained (60X magnification) using the Marianas Microscopy System (Intelligent Imaging Innovations).

### Apoptosis analysis

BCR-ABL Arf^*−/−*^ GFP^+^ cells in media with vehicle or 50–100 nM ATRA were plated in a 6-well plate at a density of 125,000 cells/mL and incubated at 37 °C, 8% CO_2_ for 48 hr, then transferred to a 15 mL falcon tube, pelleted, and the media removed. The cell pellet was washed twice with PBS, resuspended in 100 μL of Annexin-V Binding Buffer containing 10 μL Annexin-V-APC (BD Bioscience, cat#550475) and 10 μL DAPI. The sample was then vortexed and incubated for 15 min at room temperature in the dark. 150 μL of additional cold binding solution was added, and the cells are filtered through 40 μm nylon mesh and placed on ice until analysis for Annexin-V positive cells using FACS. The percentage of Annexin-V positive cells were compared by treatment groups.

### Viability assays

ATRA can bind to RARα, RARβ and RARγ but ATRA can only bind to RXRα following conversion to 9-cis retinoic acid. In order to determine the pathway through which ATRA acts to affect BCR-ABL cell viability, we tested leukemic cell viability with specific agonists for RARα (BMS753); RARβ (CD2314); RARγ (BMS961); or RXRα (CD3254), or with the pan-RXRα, β,γ, agonist bexarotene [targretin], or the pan-RXR/RAR agonist 9-cis-retinoic acid [Tocris Bioscience, Bristol, UK] (Table 1). Results were compared to those with ATRA (Sigma-Aldrich Inc., St Louis, MO, USA). Concentrations used were selected based on the known affinities (Kd) of each agonist for its receptor. 6,250 luciferase- tagged BCR-ABL *Arf*^*−/−*^ mouse cells were plated at 100 µL/well of white, clear-bottomed 96-well plate (Costar Corning Inc., Kennebunk, ME, USA) with each receptor agonist and incubated 48 hr at 37 °C, 8% CO_2_. MTT (3-(4,5-dimethylthiazol-2-yl)-2,5-diphenyltetrazolium bromide) and MTS ((3-(4,5-dimethylthiazol-2-yl)-5-(3-carboxymethoxyphenyl)-2-(4-sulfophenyl)-2H-tetrazolium) assays were used to measure metabolically active viable cell number according to the manufacturer’s protocol (Abcam, Cambridge, MA, USA). MTT and MTS assays measure cell viability by measuring mitochondria enzyme activity as a surrogate of mitochondrial function with the reaction product formation dependent on the number of metabolically active viable cells. Absorbance was measured using BioTek Synergy^TM^ 4 Hybrid Microplate Reader at OD = 490 nm.

### Data analysis

Kaplan Meier curves were generated to compare survival data between the groups and the Gehan-Breslow-Wilcoxon Test was used to determine significance. The Welch’s t-test was used to determine significant differences in ROI means between the groups. Luciferase signals of blood, spleen, and bone marrow, and ELISA data were analyzed using Mann-Whitney nonparametric test on GraphPad Prism versions 5 and 7. Blood chemistry data were analyzed using 1way ANOVA with Tukey’s multiple comparison test. Significance is calculated at p < 0.05 and outliers were determined using Grubb’s test on GraphPad QuickCalcs.

Unpaired t-tests were used to determine significance in other assay’s comparing cells treated with 1, 25(OH)_2_VD_3_ or ATRA or vehicle. Flow cytometry comparisons were done using unpaired t-tests using GraphPad Prism software.

## Supplementary information

Supplementary Information.

## Data Availability

All datasets generated for this study are available from the corresponding author upon request.
