# Supplementary material for: Role of Vitamins A and D in BCR-ABL Arf−/− Acute Lymphoblastic Leukemia
Source: Sci Rep. 2020 Feb 11;10:2359. doi: 10.1038/s41598-020-59101-4 (PMC7012907; doi:10.1038/s41598-020-59101-4)
Supplement: Supplementary file 1 — Supplementary Information. [file 41598_2020_59101_MOESM1_ESM.pdf]

Supplementary data

Role of Vitamin A and D in BCR-ABL *Arf*$^{-/-}$ Acute Lymphoblastic Leukemia

Kavya Annu[1+], Cynthia Cline[1+], Kazuto Yasuda[1+], Samit Ganguly[1+], Andrea Pesch[1+], Brittany Cooper[1], Laura Janke[2], Monique Payton[1], Kamalika Mukherjee[1], Sherri L. Surman[3], Julia L. Hurwitz[3], and Erin G. Schuetz[1*]

[1]Department of Pharmaceutical Sciences, St. Jude Children's Research Hospital, Memphis, TN, USA.

[2]Department of Pathology, St. Jude Children's Research Hospital, Memphis, TN, USA.

[3]Department of Infectious Diseases, St. Jude Children's Research Hospital, Memphis, TN, USA.

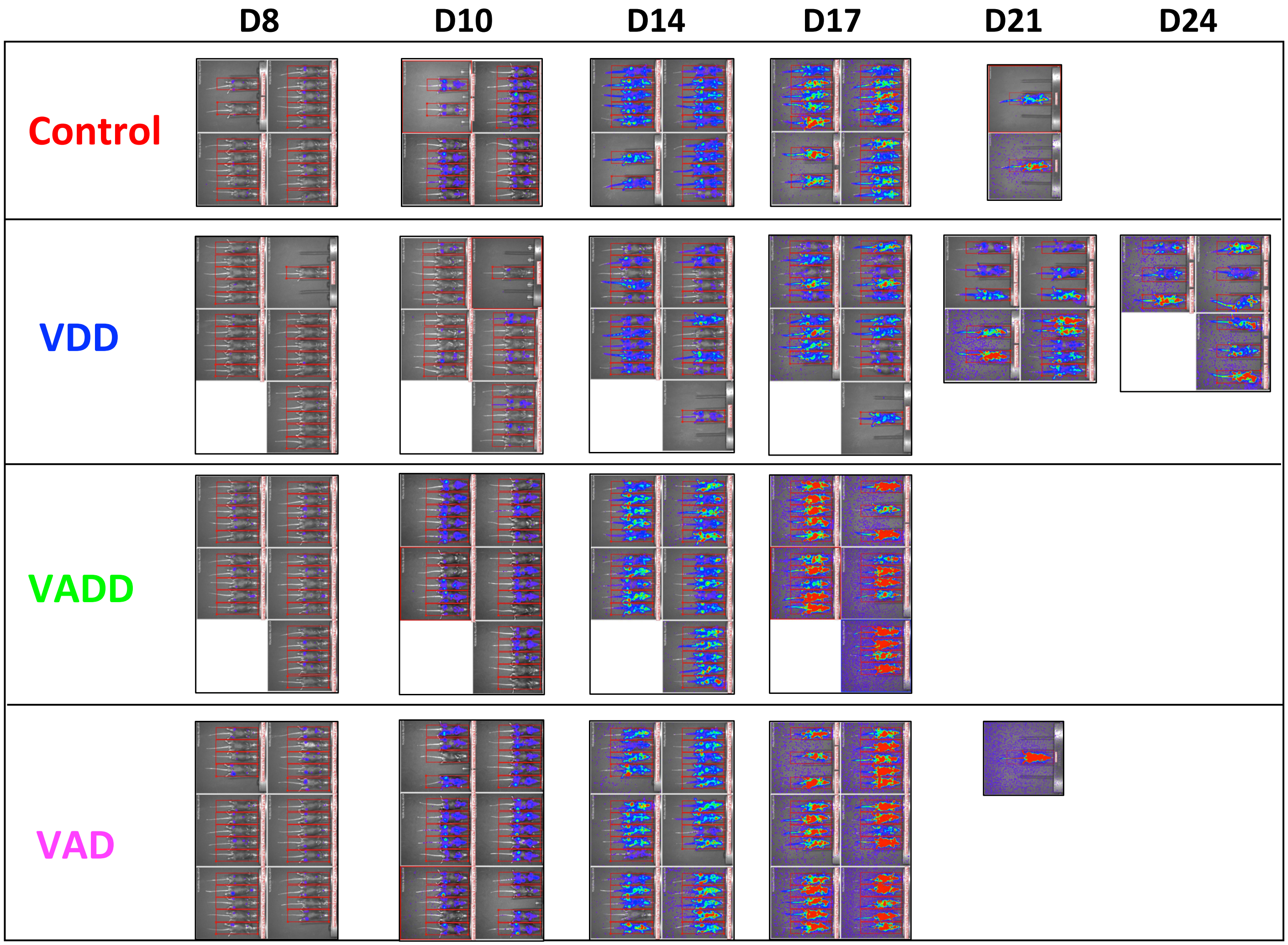

Fig S1.

BCR-ABL Luc+ ALL disease burden in representative male mice

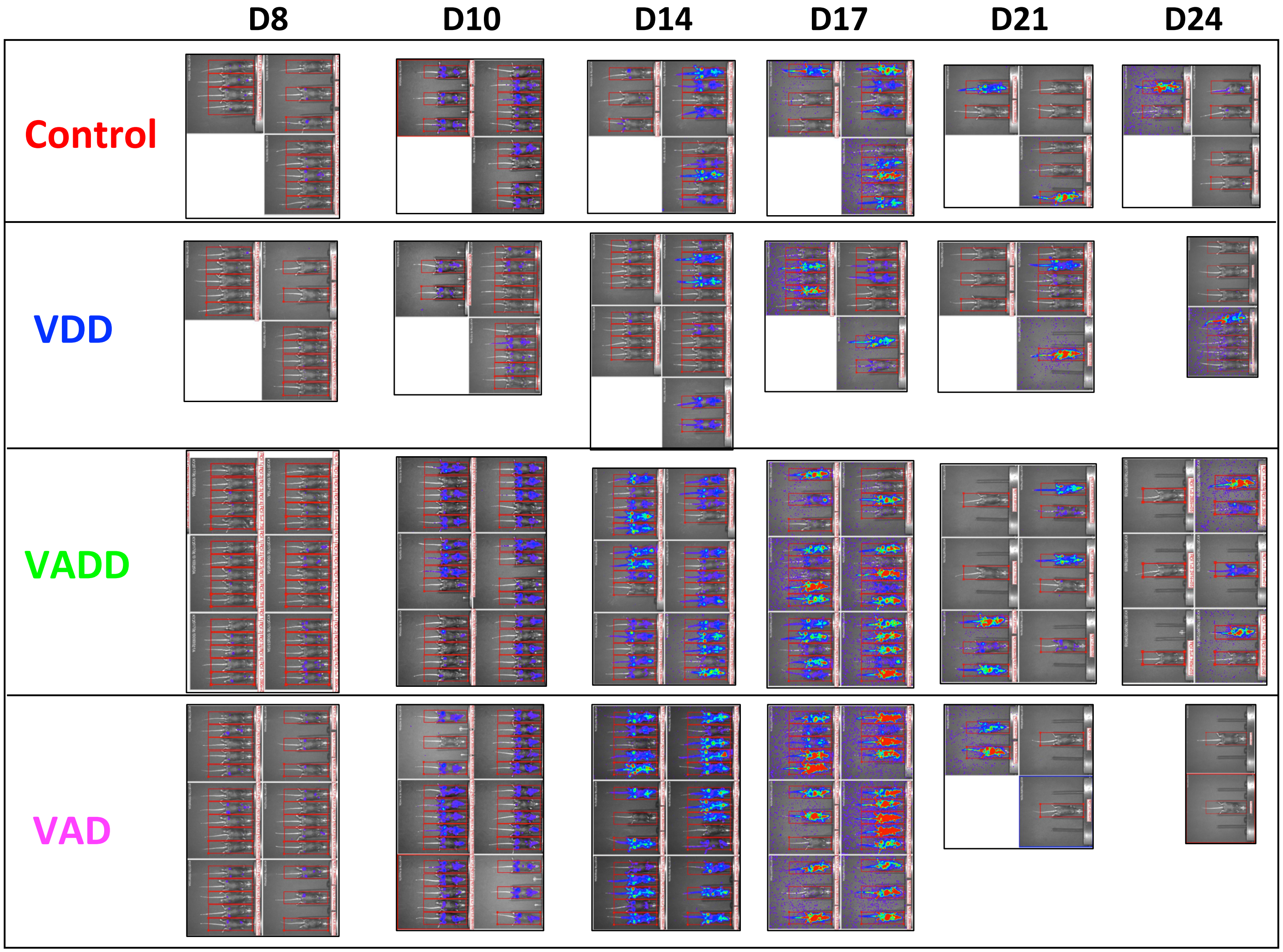

Fig S2.

BCR-ABL Luc^{+} ALL disease burden in representative female mice

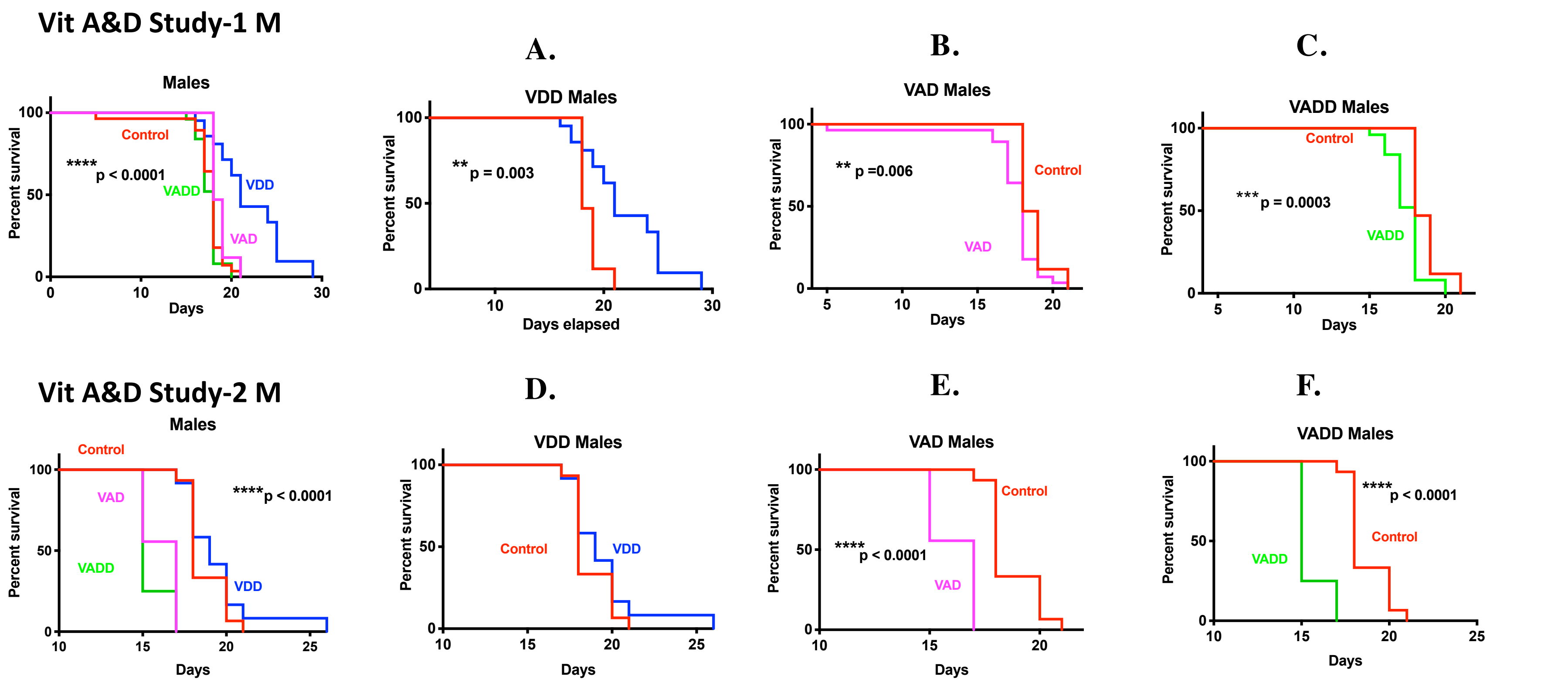

Fig S3. Kaplan Meier survival curves for males (study 1:A-C, study 2: D-F) **(A,D)** control and VDD mice; **(B,E)** control and VAD mice; and **(C,F)** control and VADD mice. The Long-Rank Mantel-cox test was used to find differences between survival curves of control and vitamin deficient groups (****p<0.0001). Individual deficient group vs. control results were compared using the Gehan-Breslow-Wilcoxon test (****p<0.000, **p<0.01).

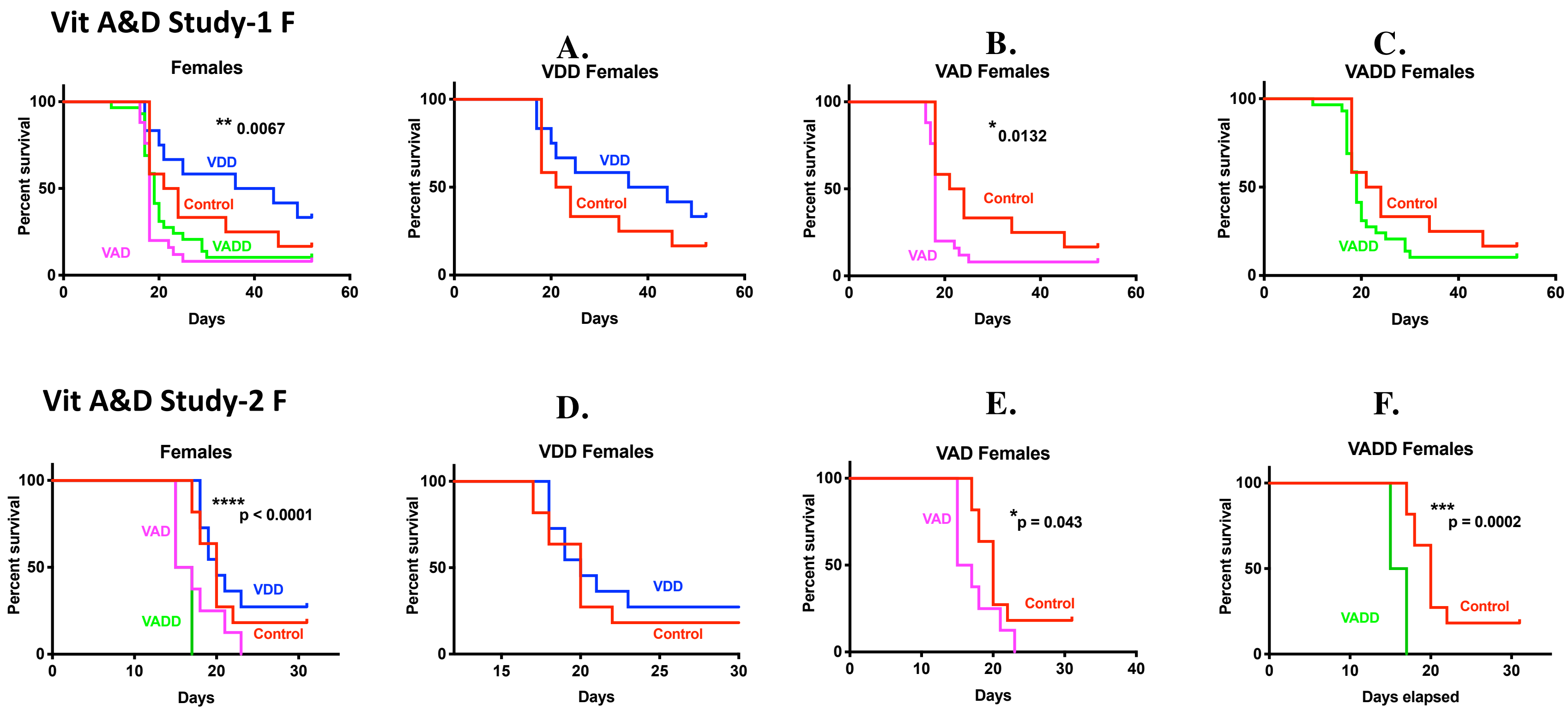

Fig S4. Kaplan Meier survival curves for females (study 1:A-C, study 2: D-F) **(A,D)** control and VDD mice; **(B,E)** control and VAD mice; and **(C,F)** control and VADD mice. The Long-Rank Mantel-cox test was used to find differences between survival curves of control and vitamin deficient groups (****p<0.0001). Individual deficient group vs. control results were compared using the Gehan-Breslow-Wilcoxon test (****p<0.000, **p<0.01).

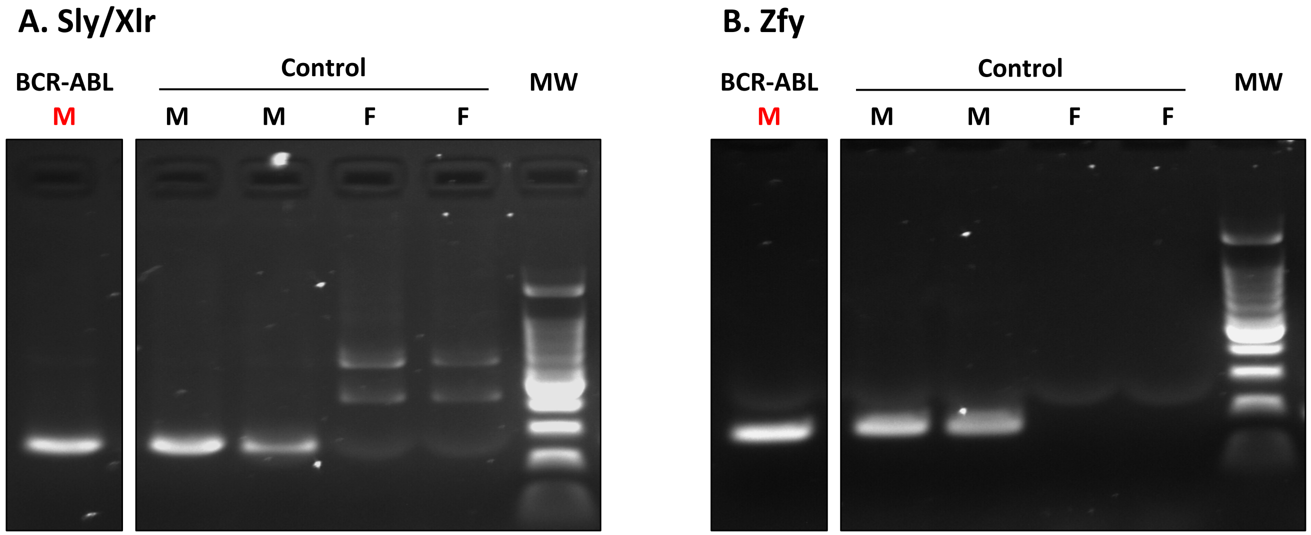

Fig. S5. Determination of mouse gender using Sly/Xlr and Zfy PCR. Genomic DNA from YY male and XY female mice were amplified with primers for **(A)** Y chromosome Sly intron 8 and **(B)** X chromosome Xlr intron 6 and Y chromosome Zfy.

Fig S5.

A. Sly/Xlr

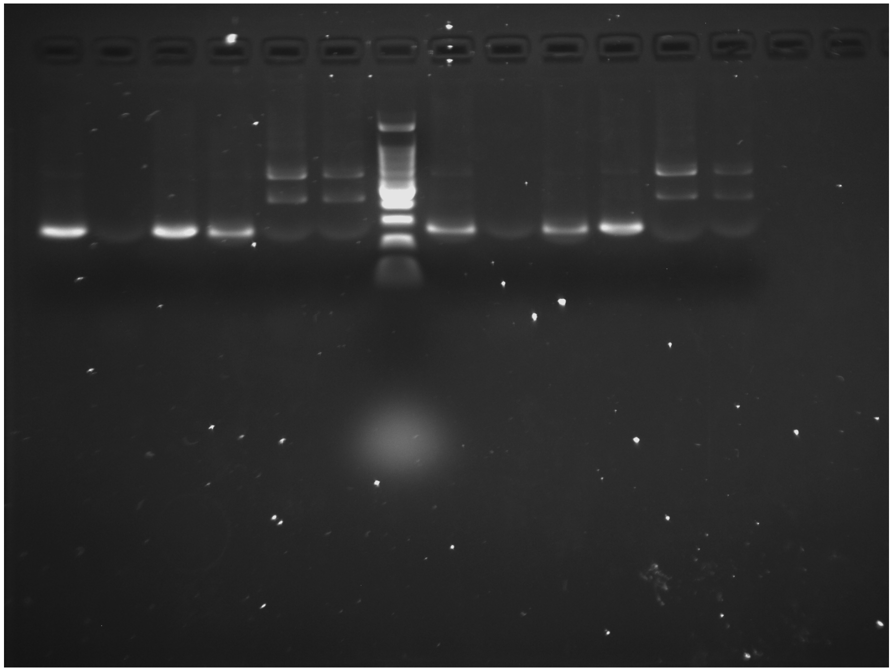

B. Zfy

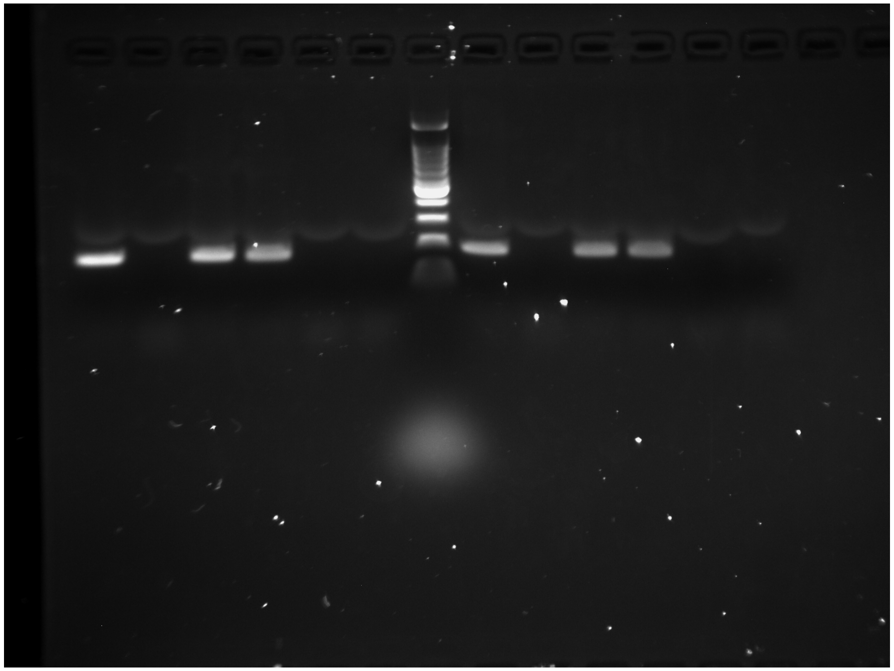

The original gel used to create Fig. S5. Lane 2 was removed due to low RNA quality of the sample, lanes 8-13 were duplicates of lane 1-6. Lanes in Fig. S5. correspond to lanes 1, 3-7 in the above respective figures.

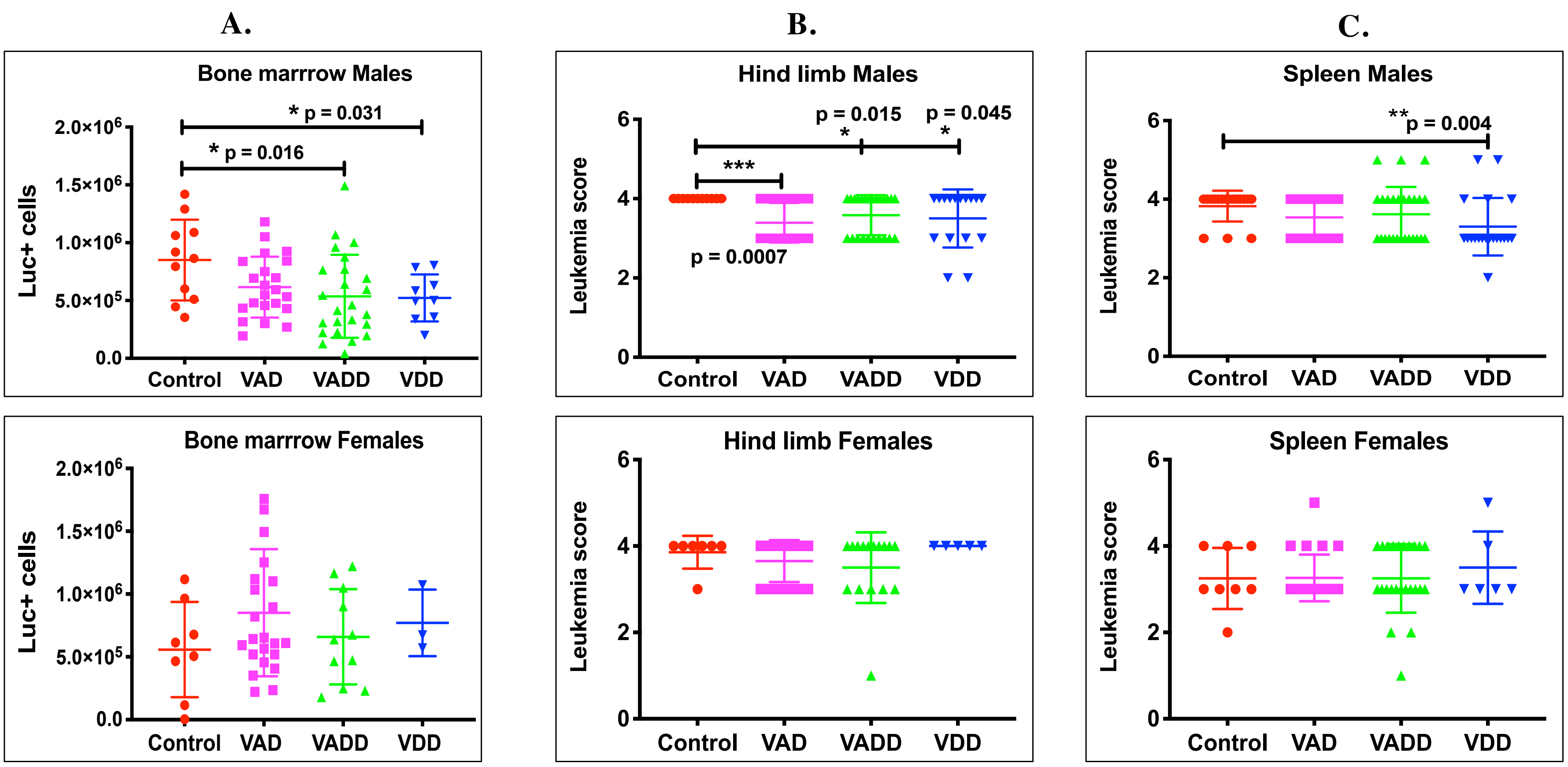

Fig S6. (A) Luminescence measure of BCR-ABL-Luc Bone marrow disease burden (M, F), **(B)** Hind Limb leukemia score (M, F), and **(C)** Spleen leukemia score (M, F) scored by Pathologist analysis of H&E slides. Mann-Whitney nonparametric test on GraphPad was used to determine significance between the groups (*p<0.05).

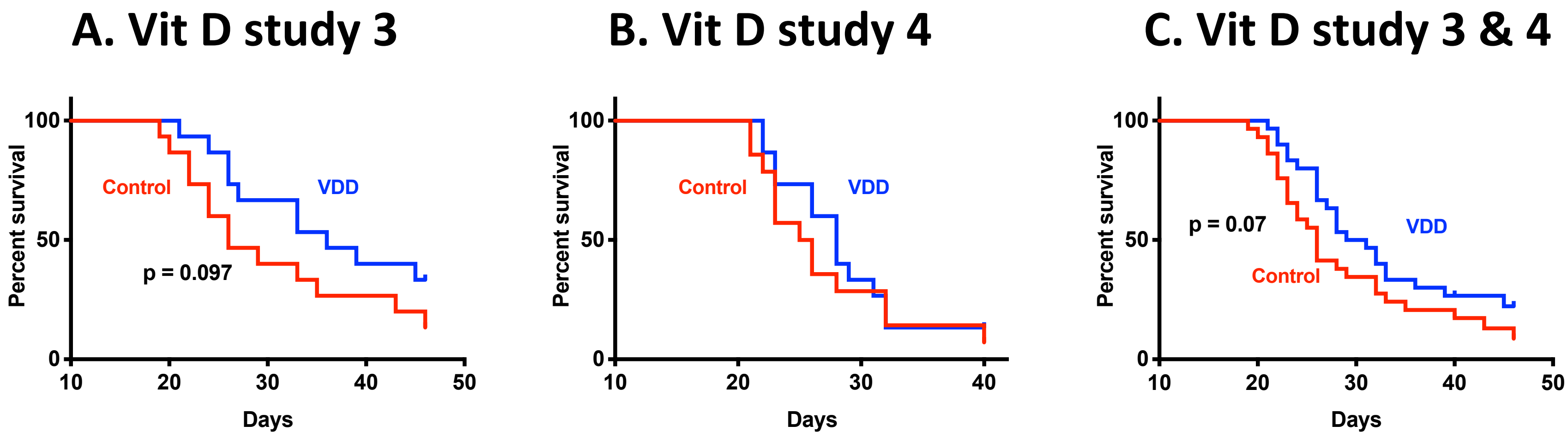

Fig S7. Kaplan Meier survival curves using male mice started on control and VDD diet beginning at weaning **(A)** study 3 - control (n=15) vs. VDD (n=15), **(B)** study 4 - control (n=15) vs. VDD (n=15), **(C)** study 3 & 4 combined. Differences between survival curves of control and VDD group was determined using the Gehan-Breslow-Wilcoxon test. The median survival for the control mice vs. VDD mice was 26 vs. 36 days (Study 3) and 26 vs. 30 days (Study 4).

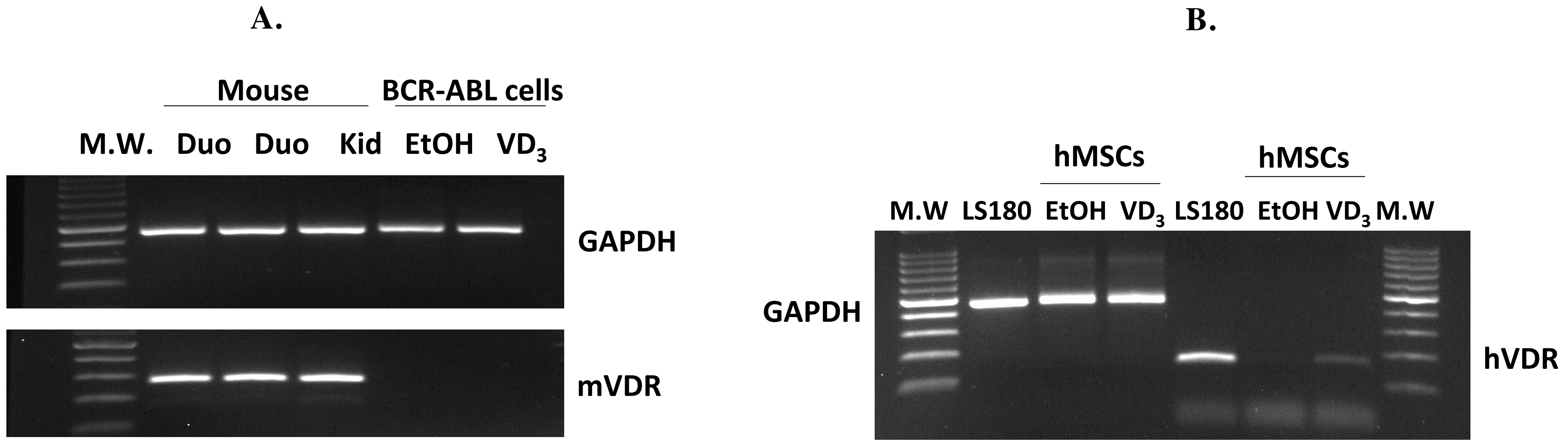

Fig S8. Determination of VDR expression using PCR. **(A)** Lane 1: M.W, Lane 2-6 showing GAPDH band at 500bp and lane 2-4 (positive control: mouse duodenum, kidney) mVDR band at 300bp, no band in lane 5-6 for mVDR in BCR-ABL ALL cells. **(B)** Lane 2-4 showing GAPDH band at 500bp and lane 5 (positive control, LS180 cells) hVDR band at 190bp, lane 6: hMSCs treated with ethanol no band of hVDR, lane 7: hMSCs treated with $1,25(OH)_2VD_3$ a band of hVDR.

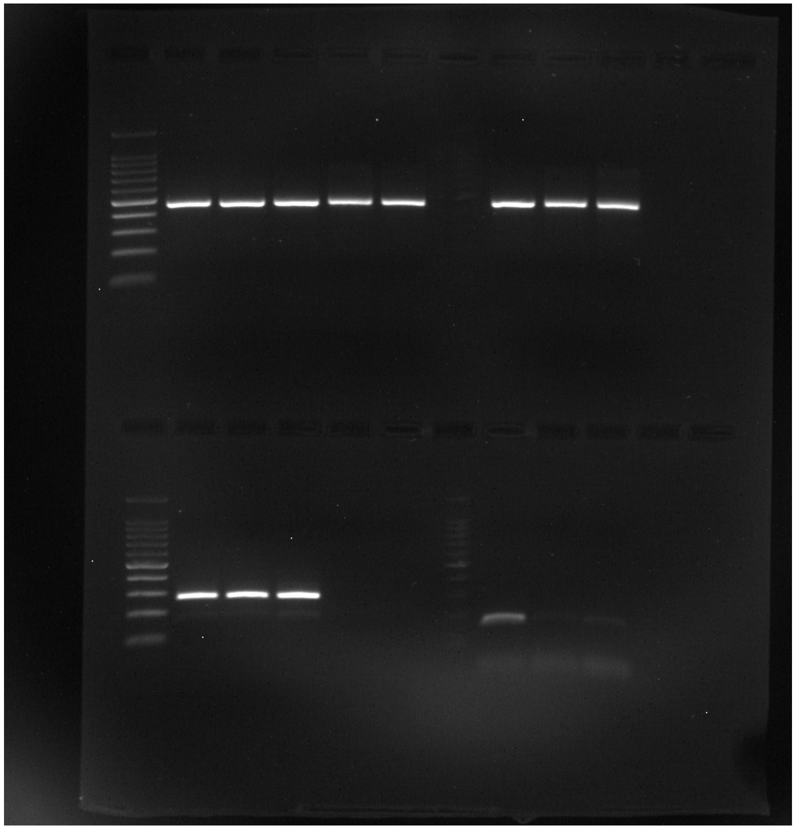

The original image used to create **Fig S8 (A).** Top gel, lane 2-6 (samples same as S8(A) for GAPDH. Bottom gel (same samples as top gel), lane 2-6 for mVDR. Lane 1 molecular weight markers. Lanes 7-10 not used for Fig S8.

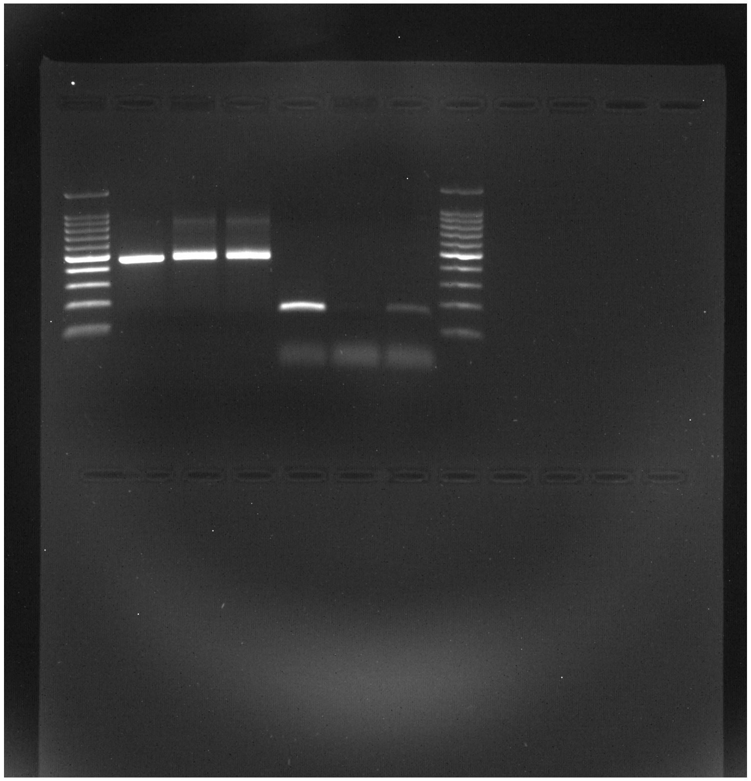

The original image used to create **Fig S8 (B).** Lane 2-4 (samples same as S8(B) for GAPDH. Lanes 5-7 (samples same as S8(B) for hVDR. Lanes 1 and 8 for molecular weight markers.

Table S1. Survival proportions of all groups per individual experiment

Males

Study 1

Dates elapsed	Control	VAD	VADD	VDD
0	100	100	100	100
5		96.43		
15			96.00	
16		89.29	84.00	95.24
17		64.29	52.00	85.71
18	47.06	17.86	8.00	80.95
19	11.76	7.14		71.43
20		3.57	0.00	61.90
21	0.00	0.00		42.86
24				33.33
25				9.52
29				0.00

Study 2

Dates elapsed	Control	VAD	VADD	VDD
0	100	100	100	100
15		55.56	25.00	
17	93.33	0.00	0.00	91.67
18	33.33			58.33
19				41.67
20	6.67			16.67
21	0.00			8.33
26				0.00

Females

Dates elapsed	Control	VAD	VADD	VDD
0	100	100	100	100
10			96.55	
16		88.00	93.10	
17		76.00	68.97	83.33
18	58.33	20.00	58.62	
19			41.38	
20			31.03	75.00
21	50.00		27.59	66.67
22		16.00		
23		12.00	24.14	
24	33.33			
25		8.00	20.69	58.33
29			13.79	
30			10.34	
34	25.00			
36				50.00
44				41.67
45	16.67			
49				33.33
52	16.67	8.00	10.34	33.33

Days elapsed	Control	VAD	VADD	VDD
0	100	100	100	100
15		50.00	50.00	
17	81.82	37.50	0.00	
18	63.64	25.00		72.73
19				54.55
20	27.27			45.45
21		12.50		36.36
22	18.18			
23		0.00		27.27
31	18.18			27.27

Table S2. Vit A & D: Serum RBP, 25-OH VD_3 values across groups (Terminal time point, Representative data)

Group	RBP (ug/ml)	25-OH VD_3 (ng/ml)
CON F	12,979	102.8
CON F	9,806	50.4
CON M	14,017	87.6
CON M	15,754	36.4
VAD F	1,739	53.6
VAD F	1,267	40.4
VAD M	1,672	36.4
VAD M	1,668	38.4
VADD F	2,658	3.6
VADD F	2,896	6.4
VADD M	2,501	9.2
VADD M	2,006	5.6
VDD F	12,896	2.4
VDD F	8,706	1.6
VDD M	17,897	3.2
VDD M	15,177	2.4

25-OH VD_3	Vitamin D Status
<12 ng/mL	Deficient
12–20 ng/mL	Insufficient
≥20 ng/mL	Sufficient

RBP	Vitamin A Status
<5,000 ng/mL	Deficient
>5,000 ng/mL	Sufficient

https://ods.od.nih.gov/factsheets/VitaminD-HealthProfessional/

Table S3.

Vit A & D: Male CBC (Terminal time point, Representative data)

BCR-ABL ALL — **No leukemia injected control**

	Control M		VAD M		VDD M		VADD M		Control M ND		VAD M ND		VDD M ND		VADD M ND	
Male	Avg	S.D	Avg	S.D	Avg	S.D	Avg	S.D	Avg	S.D	Avg	S.D	Avg	S.D	Avg	S.D
WBC (X 103/uL)	45.94	30.10	38.78	15.48	54.02	43.31	27.46	24.36	2.55	2.19			5.57	0.72	2.76	
Neutrophil (#)	18.46	9.19	17.22	7.55	20.43	14.77	12.05	12.47	0.39	0.32			1.11	0.11	0.70	
Lymphocyte (#)	15.43	13.26	10.78	4.97	19.80	17.91	8.12	5.73	1.97	1.71			4.00	0.73	1.83	
Monocyte (#)	10.14	8.16	7.07	2.93	11.39	9.41	5.22	5.04	0.19	0.17			0.47	0.11	0.23	
Eosinophil (#)	1.73	1.43	2.84	2.37	2.13	2.07	1.77	2.08	0.01	0.01			0.01	0.01	0.00	
Basophil (#)	0.19	0.21	0.87	0.78	0.26	0.32	0.31	0.41	0.01	0.01			0.00	0.00	0.00	
Nucleated RBC (#)	0.00	0.00	0.00	0.00	0.00	0.00	0.00	0.00	0.00	0.00			0.00	0.00	0.00	
Neutrophil (%)	41.99	8.07	44.76	7.82	41.00	10.31	40.31	8.99	15.22	0.51			19.89	0.62	25.19	
Lymphocyte (%)	32.74	7.56	28.37	8.85	35.37	8.02	38.74	13.97	76.98	0.74			71.46	3.83	66.20	
Monocyte (%)	21.21	5.42	18.31	2.47	19.76	6.84	16.39	6.54	7.10	0.62			8.54	3.09	8.46	
Eosinophil (%)	3.67	2.78	6.56	3.12	3.50	2.60	3.89	3.97	0.45	0.46			0.13	0.11	0.15	
Basophil (%)	0.38	0.40	2.00	1.05	0.38	0.30	0.67	0.86	0.26	0.37			0.00	0.00	0.00	
Nucleated RBC (%)	0.00	0.00	0.00	0.00	0.00	0.00	0.00	0.00	0.00	0.00			0.00	0.00	0.00	
Hematocrit (%)	33.50	2.94	28.00	4.31	30.42	2.16	28.98	2.26	31.60	1.41			31.05	2.19	33.90	
RBC (X 106/uL)	8.03	0.71	6.63	0.91	7.23	0.71	7.00	0.49	7.89	0.45			7.55	0.91	8.03	
Hemoglobin (g/dL)	12.01	1.18	10.02	1.39	10.95	0.86	10.54	0.65	12.05	0.49			11.60	0.71	12.90	
Mean Corpuscular Volume (fL)	41.72	1.38	42.12	1.02	42.23	2.48	41.39	0.86	40.05	0.49			41.25	2.05	42.20	
Mean Corpuscular Hemoglobin (pg)	14.95	0.58	15.10	0.47	15.22	0.93	15.06	0.54	15.30	0.28			15.45	0.92	16.10	
Mean Corpuscular Hemoglobin Conc(g/dL)	35.83	0.76	35.89	1.32	36.00	1.33	36.43	1.13	38.10	0.14			37.35	0.35	38.10	
Red Cell Distribution Width (%)	13.45	0.91	12.77	0.34	14.03	1.52	12.91	0.64	13.70	0.57			13.95	0.49	14.50	
RSD	5.61	0.56	5.38	0.19	5.94	0.97	5.36	0.33	5.50	0.14			5.75	0.49	6.10	
Reticulocyte (#)	14.69	17.73	4.32	5.03	25.15	31.16	9.94	12.16	9.85	12.80			8.95	12.66	14.50	
Reticulocyte (%)	0.20	0.25	0.07	0.09	0.37	0.51	0.14	0.16	0.13	0.17			0.13	0.18	0.18	
Platelet (X 103/uL)	412.80	114.23	257.33	43.81	393.08	116.43	351.13	92.87	505.50	27.58			469.50	154.86	668.00	
Mean Platelet Volume (fL)	5.68	0.39	5.54	0.35	5.76	0.49	5.24	0.38	5.00	0.00			4.85	0.07	5.00	
Platelet Distribution Width	65.15	9.56	66.86	9.29	69.36	11.66	57.03	11.27	48.90	0.42			49.80	0.57	48.60	
Plateletcrit	0.24	0.08	0.14	0.03	0.23	0.08	0.18	0.05	0.25	0.01			0.23	0.07	0.33	

Vit A & D: Male Serum chemistry (Terminal time point, Representative data)

	Control M Avg	S.D	VAD M Avg	S.D	VDD M Avg	S.D	VADD M Avg	S.D	Control M ND Avg	S.D	VAD M ND Avg	S.D	VDD M ND Avg	S.D	VADD M ND Avg	S.D
Triglyceride (mg/dl)	52.99	15.89	44.63	13.17	48.75	13.04	46.50	10.22	66.15	3.04			39.15	17.61	62.00	
HDL (mg/dl)	47.24	12.71	58.46	10.49	57.50	5.01	63.14	10.81	87.15	6.58			45.25	42.78	81.30	
LDL (mg/dl)	9.15	1.99	18.31	16.24	10.35	3.61	12.98	2.37	8.45	0.49			4.10	3.25	7.10	
Cholesterol (mg/dl)	102.82	27.20	116.00	16.24	124.00	8.76	114.88	10.26	139.50	3.54			73.00	70.71	126.00	

ND = No leukemia injected control

One-way ANOVA with Tukey's multiple comparison test; Red and Green fonts indicate increased and decreased levels between BCR-ABL ALL mice (control vs. VAD, VDD AND VADD).

Table S4.

Vit A & D: Female CBC (Terminal time point, Representative data)

BCR-ABL ALL | **No leukemia injected control**

	Control F		VAD F		VDD F		VADD F		Control F ND		VAD F ND		VDD F ND		VADD F ND	
Female	Avg	S.D	Avg	S.D	Avg	S.D	Avg	S.D	Avg	S.D	Avg	S.D	Avg	S.D	Avg	S.D
WBC (X 103/uL)	49.61	33.79	29.79	23.29	25.47	10.02	25.60	19.71	2.79	0.44	2.28		3.36	1.73	2.67	0.33
Neutrophil (#)	17.40	13.42	10.90	7.51	10.50	6.42	8.36	6.56	0.63	0.23	0.40		0.54	0.16	0.80	0.06
Lymphocyte (#)	18.25	13.25	11.90	13.52	9.09	4.81	11.03	9.64	1.93	0.19	1.62		2.51	1.48	1.69	0.27
Monocyte (#)	11.78	7.55	6.17	4.86	4.98	2.51	5.63	4.28	0.24	0.01	0.24		0.31	0.08	0.18	0.01
Eosinophil (#)	1.97	3.34	0.70	0.72	0.82	1.06	0.44	0.46	0.00	0.00	0.01		0.01	0.01	0.00	0.00
Basophil (#)	0.21	0.32	0.12	0.14	0.08	0.14	0.14	0.19	0.00	0.00	0.01		0.00	0.00	0.00	0.00
Nucleated RBC (#)	0.00	0.00	0.00	0.00	0.00	0.00	0.00	0.00	0.00	0.00	0.00		0.00	0.00	0.00	0.00
Neutrophil (%)	35.92	7.94	41.52	12.72	40.63	14.56	34.24	7.75	22.09	4.90	17.64		17.12	4.02	29.91	1.22
Lymphocyte (%)	37.99	6.48	35.55	12.05	36.37	13.17	44.40	8.94	69.22	4.00	71.25		72.91	6.71	63.20	2.47
Monocyte (%)	22.99	8.91	19.92	4.59	20.03	5.89	19.70	7.43	8.60	0.88	10.63		9.74	2.54	6.76	1.30
Eosinophil (%)	2.80	3.34	2.56	1.98	2.70	2.93	1.30	0.86	0.04	0.06	0.26		0.18	0.07	0.05	0.06
Basophil (%)	0.31	0.30	0.45	0.49	0.27	0.39	0.36	0.42	0.06	0.08	0.23		0.07	0.09	0.10	0.01
Nucleated RBC (%)	0.00	0.00	0.00	0.00	0.00	0.00	0.00	0.00	0.00	0.00	0.00		0.00	0.00	0.00	0.00
Hematocrit (%)	33.92	1.52	29.83	4.75	32.39	2.47	27.91	4.56	37.50	0.71	33.90		38.60	0.14	35.05	6.01
RBC (X 106/uL)	8.00	0.54	6.89	1.12	7.91	0.67	6.65	1.09	9.08	0.36	8.14		9.57	0.08	8.46	1.49
Hemoglobin (g/dL)	12.10	0.66	10.48	1.73	11.58	1.04	9.96	1.59	14.20	0.42	12.70		14.80	0.28	13.35	2.19
Mean Corpuscular Volume (fL)	42.47	1.95	43.34	1.04	41.03	1.32	42.00	1.20	41.30	0.85	41.70		40.35	0.21	41.45	0.21
Mean Corpuscular Hemoglobin (pg)	15.13	0.71	15.21	0.36	14.65	0.54	14.99	0.37	15.65	0.21	15.60		15.50	0.42	15.80	0.14
Mean Corpuscular Hemoglobin Conc(g/dL)	35.67	0.71	35.13	0.85	35.73	1.15	35.69	1.40	37.90	0.42	37.50		38.35	0.92	38.10	0.28
Red Cell Distribution Width (%)	13.89	1.31	13.13	1.19	13.63	0.42	13.11	0.31	13.70	0.00	12.90		14.25	0.21	13.45	0.07
RSD	5.92	0.82	5.69	0.55	5.59	0.19	5.50	0.23	5.65	0.07	5.40		5.75	0.07	5.60	0.00
Reticulocyte (#)	23.69	24.76	15.35	14.55	36.50	17.83	19.31	19.72	0.00	0.00	3.30		2.85	4.03	15.55	21.99
Reticulocyte (%)	0.31	0.33	0.24	0.23	0.47	0.24	0.29	0.28	0.00	0.00	0.04		0.03	0.04	0.21	0.30
Platelet (X 103/uL)	419.44	140.62	299.63	58.07	345.88	151.18	313.14	48.27	497.50	27.58	519.00		586.00	22.63	482.50	23.33
Mean Platelet Volume (fL)	5.67	0.33	5.29	0.22	5.64	0.33	5.34	0.24	4.85	0.07	5.10		4.80	0.00	4.80	0.00
Platelet Distribution Width	68.77	10.88	55.84	5.80	58.30	13.64	60.99	14.57	48.55	0.64	50.60		49.40	0.00	50.00	0.85
Plateletcrit	0.24	0.09	0.16	0.03	0.20	0.09	0.17	0.02	0.24	0.02	0.27		0.28	0.01	0.23	0.01

Vit A & D: Female Serum chemistry (Terminal time point, Representative data)

	Control F Avg	Control F S.D	VAD F Avg	VAD F S.D	VDD F Avg	VDD F S.D	VADD F Avg	VADD F S.D	Control F ND Avg	Control F ND S.D	VAD F ND Avg	VAD F ND S.D	VDD F ND Avg	VDD F ND S.D	VADD F ND Avg	VADD F ND S.D
Triglyceride (mg/dl)	44.54	7.41	44.53	7.23	36.33	12.51	44.97	9.08	82.50	28.57	30.90		60.20	25.17	43.20	2.55
HDL (mg/dl)	50.76	6.43	44.43	12.76	34.88	10.18	46.81	6.51	53.60	3.68	54.00		51.55	0.07	56.55	2.90
LDL (mg/dl)	13.32	5.23	13.00	4.60	9.53	3.54	12.70	3.27	12.95	0.49	7.30		10.25	0.64	8.95	0.35
Cholesterol (mg/dl)	113.60	7.06	94.00	18.05	80.25	25.05	99.29	15.10	102.50	10.61	86.00		89.00	0.00	90.50	3.54

ND = No leukemia injected control

One-way ANOVA with Tukey's multiple comparison test; Green font indicates decreased levels between BCR-ABL ALL mice (control vs. VAD, VDD AND VADD).
